# Gut Commensal *Bacteroidetes* Encode a Novel Class of Vitamin B_12_-Binding Proteins

**DOI:** 10.1128/mbio.02845-21

**Published:** 2022-03-01

**Authors:** E. E. Putnam, J. Abellon-Ruiz, B. J. Killinger, J. J. Rosnow, A. G. Wexler, E. Folta-Stogniew, A. T. Wright, B. van den Berg, A. L. Goodman

**Affiliations:** a Department of Microbial Pathogenesis and Microbial Sciences Institute, Yale University School of Medicine, New Haven, Connecticut, USA; b Biosciences Institute, The Medical School, Newcastle University, Newcastle upon Tyne, United Kingdom; c Biological Sciences Division, Pacific Northwest National Laboratory, Richland, Washington, USA; d The Gene and Linda Voiland School of Chemical Engineering and Bioengineering, Washington State University, Pullman, Washington, USA; e W. M. Keck Biotechnology Resource Laboratory, Yale University School of Medicine, New Haven, USA; Duchossois Family Institute

**Keywords:** *Bacteroides*, microbiome, vitamin B_12_

## Abstract

Human gut commensal *Bacteroidetes* rely on multiple transport systems to acquire vitamin B_12_ and related cobamides for fitness in the gut. In addition to a set of conserved transport proteins, these systems also include a diverse repertoire of additional proteins with unknown function. Here, we report the function and structural characterization of one of these proteins, BtuH, which binds vitamin B_12_ directly via a C-terminal globular domain that has no known structural homologs. This protein is required for efficient B_12_ transport and competitive fitness in the gut, demonstrating that members of the heterogeneous suite of accessory proteins encoded in *Bacteroides* cobamide transport system loci can play key roles in vitamin acquisition.

## INTRODUCTION

The gut microbiome is implicated in many aspects of health and disease (1). These complex communities are primarily composed of bacteria from four phyla: *Bacteroidetes*, *Firmicutes*, *Proteobacteria*, and *Actinobacteria* (1). Members of the phylum *Bacteroidetes* can comprise more than 60% of these microbial communities (2) and are known for their ability to transform intractable carbon sources into biomass and short-chain fatty acids (3). In addition to these carbon sources, these microbes also depend on a series of small molecule cofactors acquired from a combination of biosynthesis and capture from host diet and other microbes (4). Although these metabolic exchanges likely play a key role in shaping microbial interactions in the gut, the underlying mechanisms are not well understood.

Vitamin B_12_-like small molecules (cobamides) provide an example of the importance of enzyme cofactors in determining commensal fitness in the gut and the elaborate mechanisms that gut commensals use to obtain these small molecules (4, 5). Although gut *Bacteroidetes* encode multiple cobamide-dependent enzymes, they generally lack the ability to synthesize these molecules *de novo* from simple precursors (4, 6). Instead, these organisms encode various forms of incomplete cobamide salvage and remodeling pathways and express dedicated systems for transport of cobamides from the extracellular environment (4, 6). Although gut *Bacteroidetes* typically encode cofactor-independent homologs of many cobamide-dependent enzymes and possess multiple cobamide transport systems, inactivation of single transporters can determine commensal fitness in a diet- and community-dependent manner (4, 5).

The vitamin B_12_ transport system from Escherichia coli is a model system for cobamide acquisition and other forms of TonB-dependent transport. In E. coli, vitamin B_12_ crosses the outer membrane via the TonB-dependent transporter BtuB (7–9). The vitamin is bound by BtuF in the periplasm and is transported across the inner membrane by the ABC-type transporter BtuCD (7, 10). These genes are encoded in separate operons with other genes that are not involved in B_12_ transport (11). In contrast, human gut *Bacteroidetes* typically possess multiple cobamide transport loci that include *btuBFCD* homologs in addition to a diverse collection of other genes with no homologs in E. coli (12). Only one of these accessory genes, *btuG*, is universally conserved across these loci; the corresponding protein is required for efficient cobamide transport (13). Whether the numerous additional proteins encoded in these loci are involved in cobamide transport is unknown.

Here, we use a vitamin B_12_-affinity based probe (B_12_-ABP) (14, 15) to capture vitamin B_12_-binding proteins in the human gut microbe Bacteroides thetaiotaomicron. This proteomic screen identified multiple novel B_12_-binding proteins, including three without previously known function that share a domain. We demonstrate that a representative of this family, BtuH2, binds B_12_ directly and is required for efficient B_12_ transport *in vitro* and for fitness in the gut of gnotobiotic mice. The X-ray crystal structure of BtuH2 bound to B_12_ demonstrates that the protein consists of several N-terminal Ig-like domains and a C-terminal globular domain that binds B_12_ and has no known structural homologs. Together, these results suggest that the heterogeneous suite of proteins encoded in *Bacteroidetes* cobamide transport loci includes cobamide-binding components that contribute to the function of these systems.

## RESULTS

### *Bacteroides thetaiotaomicron* encodes novel vitamin B_12_-binding proteins.

**FIG 1:**
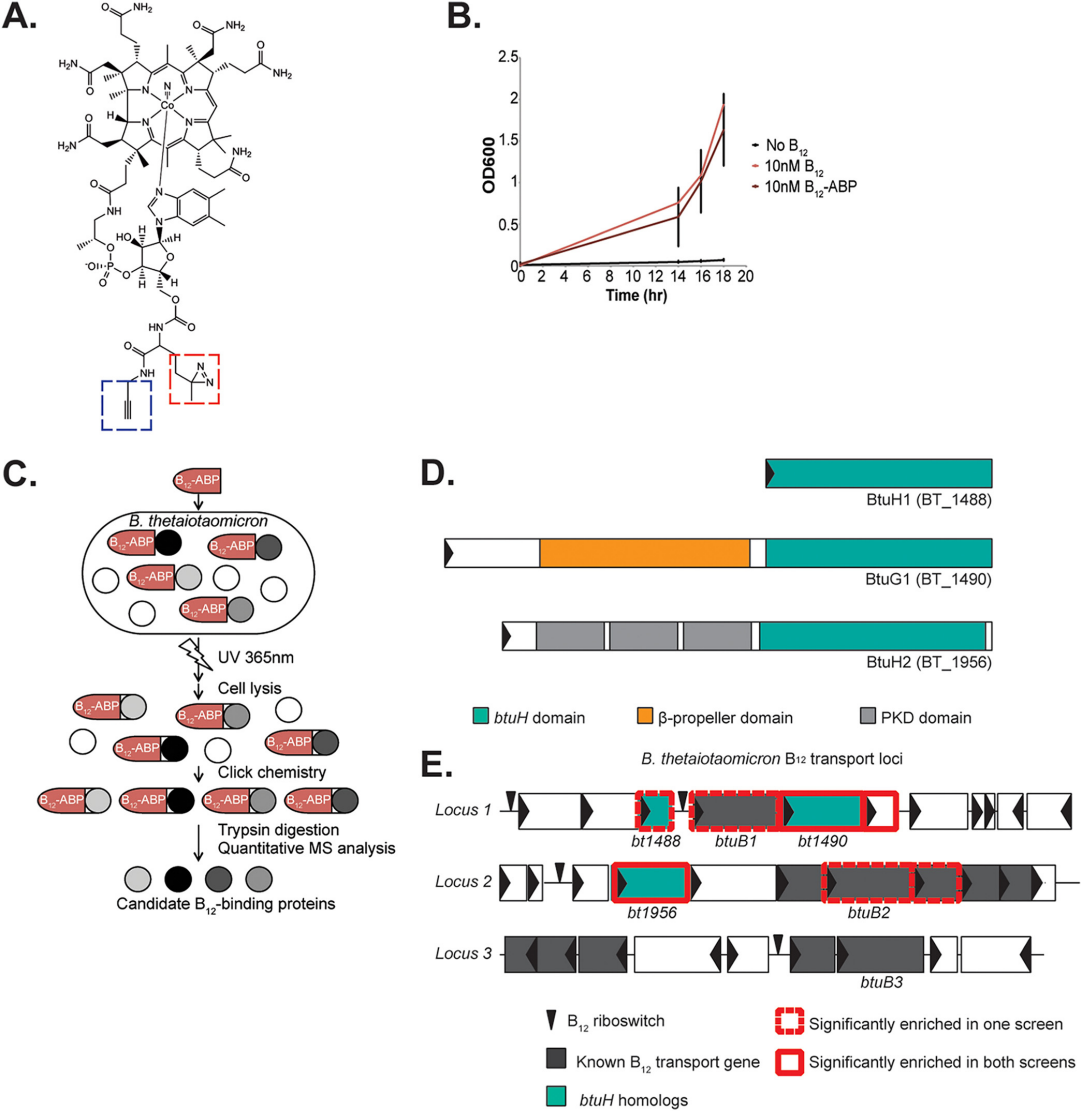
An affinity-based probe captures vitamin B_12_-binding proteins in B. thetaiotaomicron. (A) The structure of B_12_-ABP. This probe contains the native vitamin B_12_ molecule with two attachments: a diazirine (red box) for irreversible photo-cross-linking to adjacent proteins and an alkyne (blue box) for click chemistry for reporting probe binding. (B) B. thetaiotaomicron growth in minimal medium lacking methionine and containing 10 nM vitamin B_12_, 10 nM B_12_-ABP, or no B_12_. Error bars represent standard deviation of biological triplicate cultures. (C) Identification of B_12_-binding proteins using B_12_-ABP. (D) Domain organization of three uncharacterized B_12_-binding proteins. BtuH domains are shown in teal, β-propeller domains in orange, and PKD domains in gray. (E) B. thetaiotaomicron encodes three vitamin B_12_ transport loci. Red outlines denote genes encoding proteins captured by B_12_-ABP in the screen. Genes encoding previously characterized B_12_ transport proteins are shown in gray; genes encoding three uncharacterized proteins that share a region of homology at the C terminus are shown in teal.

To better understand how B. thetaiotaomicron acquires cobamides, we used B_12_-ABP (Fig. 1A) (14) to identify cobamide-binding proteins. B. thetaiotaomicron encodes only the B_12_-dependent methionine synthase MetH and is therefore dependent on B_12_ for growth when methionine is absent. Under these conditions, B. thetaiotaomicron grew at equivalent rates in the presence of B_12_ or B_12_-ABP, confirming that this organism can transport and utilize the probe as a vitamin (Fig. 1B). We next cultured this strain to mid-log phase in the presence of B_12_-ABP or unlabeled B_12_, exposed the cultures to UV irradiation, enriched and digested all probe-bound proteins, and identified these peptides by mass spectrometry-based proteomics (Fig. 1C). We conducted this screen in two independent experiments, with and without alkylation in the sample preparation step, each in biological quadruplicate. Proteins significantly enriched in the presence of the probe but not in the presence of unlabeled vitamin B_12_ are reported (Table 1 [proteins significantly enriched in both independent experiments]; see also Table S1 [proteins significantly enriched in one or both independent experiments] in the supplemental material).

**TABLE 1:** Proteins significantly enriched in the presence of the probe but not in the presence of unlabeled vitamin B_12_ in both independent replicates of the B_12_-ABP screen

Gene ID	Annotation	Avg log_2_ fold change in peptide abundance^*a*^		Comments	Source or reference
		Screen 1	Screen 2		
BT_0180	MetH, 5-methyltetrahydrofolate-homocysteine methyltransferase	3.37037206	5.10351237	Requires methylcobalamin as a cofactor	16
BT_0418	Outer membrane porin F precursor	0.67090931	0.56612023		
BT_0640	Fe-S oxidoreductase family 2	3.11646725	5.56040882	Contains a B_12_ binding motif	12
BT_0789	Malonyl CoA-acyl carrier protein transacylase	1.77261681	0.84153956		
BT_1375	Aspartokinase	1.33404098	1.38680513		
BT_1490	Putative cell surface protein	5.60091178	1.9867224	BtuG1 in B_12_ transport locus 1	13
BT_1491	Conserved hypothetical protein	8.84129263	5.83415114	Located in B_12_ transport locus 1	12
BT_1956	Putative cell surface protein	5.86139746	1.53022108	BtuH2 in B_12_ transport locus 2	This study
BT_1998	Anaerobic ribonucleoside-triphosphate reductase	3.65977591	2.8157347		
BT_2079	Peptidyl-prolyl *cis-trans* isomerase	1.6741641	2.84841207		
BT_2145	Ribonucleoside-diphosphate reductase alpha chain	0.97950531	2.98266119	Requires adenosylcobalamin as a cofactor	26, 27
BT_2419	Putative GMP synthase [glutamine-hydrolyzing]	0.91873212	0.97976994		
BT_2797	Adenosylhomocysteinase	1.05377016	1.33389028		
BT_3841	FKBP-type peptidyl-prolyl *cis-trans* isomerase (trigger factor)	0.79644085	4.87735737		
BT_4307	Putative glycogen synthase	2.61230509	1.05085244		
BT_4612	Hypothetical protein	2.29627437	1.40787681		

a B_12_-ABP versus unlabeled B_12_.

10.1128/mBio.02845-21.8 TABLE S1 Proteins enriched from B. thetaiotaomicron by B_12_-ABP. The average fold change for peptide abundance is given (B_12_-ABP versus unlabeled vitamin B_12_) only for peptides that are counted as statistically significant hits in at least one replicate of the screen. Download Table S1, XLSX file, 0.01 MB. Copyright © 2022 Putnam et al. 2022 Putnam et al. https://creativecommons.org/licenses/by/4.0/ This content is distributed under the terms of the Creative Commons Attribution 4.0 International license.

This screen successfully identified several known cobamide-binding proteins in one or both independent experiments (Table 1; see also Table S1). These include enzymes which require vitamin B_12_ as a cofactor (e.g., methionine synthase, ribonucleoside-diphosphate reductase, and methylmalonyl-CoA mutase) and homologs of the B_12_ transport proteins BtuB, BtuF, and BtuG (12, 16, 17). Together, these results suggest that this approach can successfully identify B_12_-binding proteins in B. thetaiotaomicron.

Sixteen proteins were significantly enriched in the presence of B_12_-ABP in both independent replicates of the screen (Table 1; see also Table S1). Two of these proteins (BT_1490 and BT_1956) share a region of homology of approximately 270 amino acids at their C termini (Fig. 1D); the protein sequence in this region of homology does not contain any known vitamin B_12_-binding motif and does not have any predicted domains or structures. B. thetaiotaomicron encodes a third protein, BT_1488, which shares this region of homology and was significantly enriched in one of two independent B_12_-ABP pulldown experiments (Fig. 1D; see also Table S1). Vitamin B_12_ binding by BT_1490 may be explained by its N-terminal β-propeller domain, as in BtuG proteins (13); however, BT_1956 and BT_1488 do not encode this domain and instead carry three predicted polycystic kidney disease (PKD) domains at the N terminus (BT_1956) or lack an additional N-terminal domain entirely (BT_1488) (Fig. 1D). Each of these proteins is encoded within previously identified cobamide transport loci (designated locus 1 [*bt1486-1491*], locus 2 [*bt1949-1957*], and locus 3 [*bt2090-2101*]) (12). These loci contain cobamide transport genes and cobamide-responsive riboswitches organized in one or more operons (Fig. 1E). Following existing nomenclature, we designated *bt1488* as *btuH1* and *bt1956* as *btuH2*. *Bt1490* was previously named *btuG1* based on its N-terminal β-propeller domain (13). We focused our further studies on BtuH2 because the gene for this protein is encoded in a previously characterized cobamide transport locus (locus 2) (5, 12, 13) and is the only representative of the conserved C-terminal *btuH* domain encoded in this locus.

To confirm the results of the screen, we expressed BtuH2 in E. coli and measured vitamin B_12_ (cyanocobalamin) binding of the purified protein using surface plasmon resonance (SPR). Indeed, this protein binds B_12_ directly with a *K_D_* of 6.41 × 10^−12^ M (*k*_ON_ = 1.2 × 10^9^ M^−1^ s^−1^, *k*_OFF_ = 8.61 × 10^−5^ s^−1^). Although these numbers exceed the limits of the BiaCore instrument used for SPR and the absolute values should be interpreted with caution, the SPR results indicate a strong and direct interaction between BtuH2 and vitamin B_12_.

### BtuH2 provides a fitness benefit under vitamin B_12_ limiting conditions *in vitro* and *in vivo* and influences intracellular vitamin B_12_ levels.

**FIG 2:**
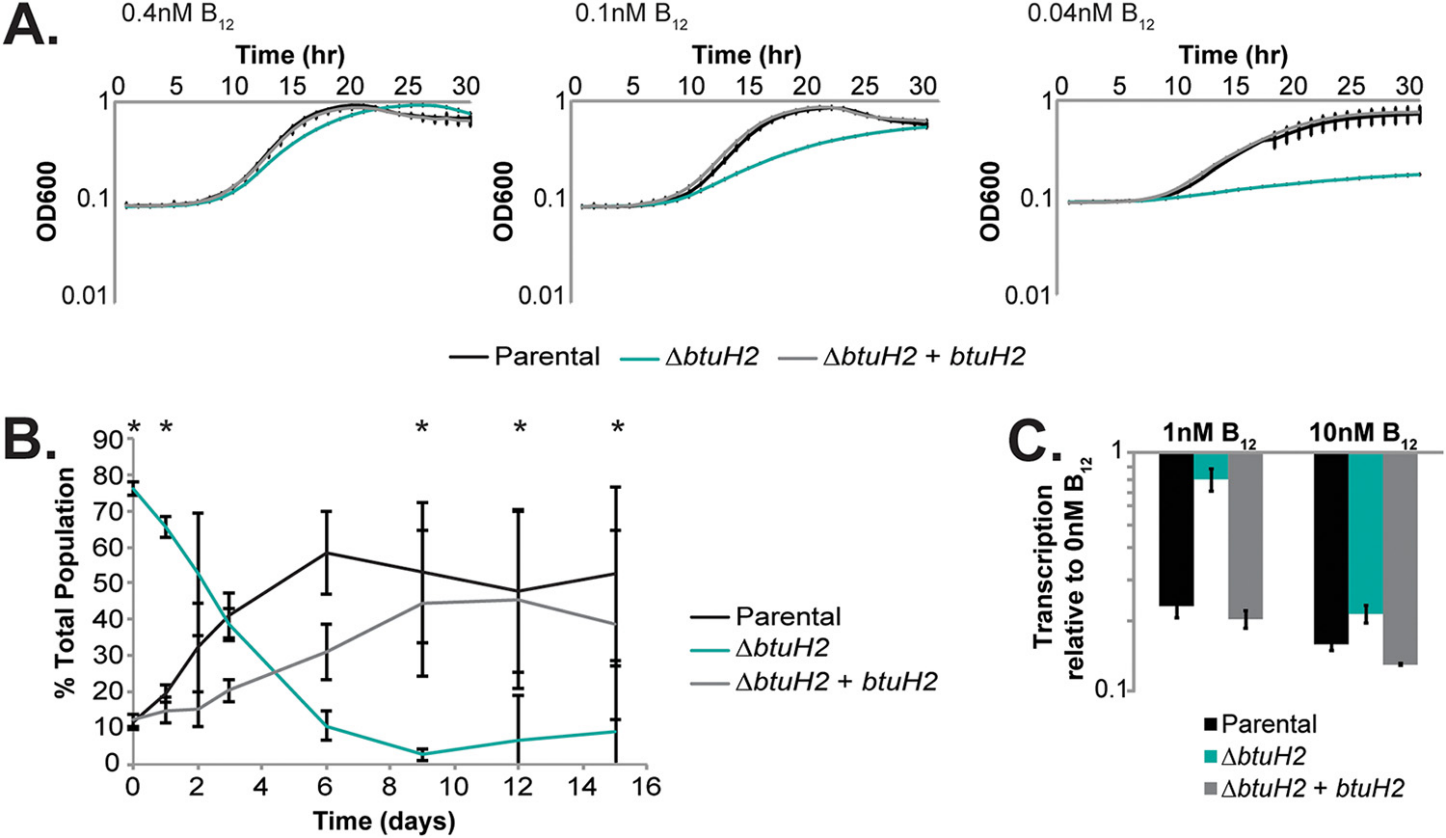
BtuH2 contributes to B. thetaiotaomicron fitness in vitamin B_12_-limiting conditions *in vitro* and during colonization *in vivo*, and is required for B_12_ transport. (A) BtuH2 is required for growth of the parental B. thetaiotaomicron strain in B_12_-limiting conditions. Error bars represent standard deviation (*n* = 4 cultures). (B) Competitive fitness of parental, Δ*btuH2* mutant, and complemented strains in germfree mice. Error bars represent standard deviation (*n* = 5 mice, repeated measure analysis of variance with the Tukey *post hoc* for pairwise comparisons of both parental and complemented strains compared to the Δ*btuH2* mutant (*, *P* < 0.05). (C) Δ*btuH2* mutants fail to repress B_12_-dependent riboswitches in the presence of 1 nM extracellular B_12_. Expression of the first gene downstream of a cobamide-dependent riboswitch (*bt1915*) was assessed by qRT-PCR.

Because B. thetaiotaomicron encodes three cobamide transport loci and three BtuH homologs, we conducted all subsequent experiments in a simplified genetic background, referred to as the parental strain, which lacks cobamide transport loci 1 and 3 and therefore encodes only a single BtuH homolog (BtuH2) (12, 13). To assess whether BtuH2 is important for B. thetaiotaomicron fitness under conditions in which vitamin B_12_ is required for growth, we cultured parental, Δ*btuH2*, and complemented strains in minimal medium lacking methionine. While the parental and complemented strains reach equivalent growth rates and final cell densities in the presence of 0.1 nM B_12_, an isogenic Δ*btuH2* mutant shows a significant growth delay under these conditions; at 0.04 nM vitamin B_12_, both growth rates and final cell densities are dependent on *btuH2* (Fig. 2A).

Inactivation of cobamide transport locus 2 compromises B. thetaiotaomicron fitness in the mouse gut (5, 12). In germfree Swiss Webster mice, parental and complemented strains rapidly outcompeted a Δ*btuH2* strain, suggesting that BtuH2 also contributes to the function of the encoded transport machinery in the gut environment (Fig. 2B).

Cobamide-dependent riboswitches repress downstream gene expression in the presence of the vitamin and serve as biosensors of intracellular B_12_ concentration (5, 18). Notably, the Δ*tdk* mutant and parental strains repress expression of B_12_ riboswitch-regulated genes at extracellular B_12_ concentrations of 1 nM (Fig. 2C; see also Fig. S1), while the Δ*btuH2* mutant fails to repress these genes until extracellular B_12_ concentrations reach 10 nM, consistent with a role for BtuH2 in B_12_ transport (Fig. 2C). Together, these studies suggest a critical role for BtuH2 in vitamin B_12_ transport *in vitro* and *in vivo*.

10.1128/mBio.02845-21.2 FIG S1 Expression of genes downstream of a predicted riboswitch decreases in the presence of B_12_. (A) The gene *bt1915* is encoded immediately downstream from a predicted B_12_-responsive riboswitch. Using the B. thetaiotaomicron Δ*tdk* strain, we assessed transcription of *bt1915* in the presence of 0, 1, and 10 nM B_12_ using qRT-PCR. Transcription levels are normalized to the 0nM B_12_ condition. Error bars represent standard deviations. (B) A transcriptional reporter was constructed using the predicted riboswitch upstream of *bt1915*, fused to the reporter *nanoluc*. Luminescence levels are normalized to the 0 nM B_12_ condition. Error bars represent standard deviations. Download FIG S1, TIF file, 1.2 MB. Copyright © 2022 Putnam et al. 2022 Putnam et al. https://creativecommons.org/licenses/by/4.0/ This content is distributed under the terms of the Creative Commons Attribution 4.0 International license.

### BtuH2 is a membrane-associated protein with a predicted lipoprotein export sequence.

**FIG 3:**
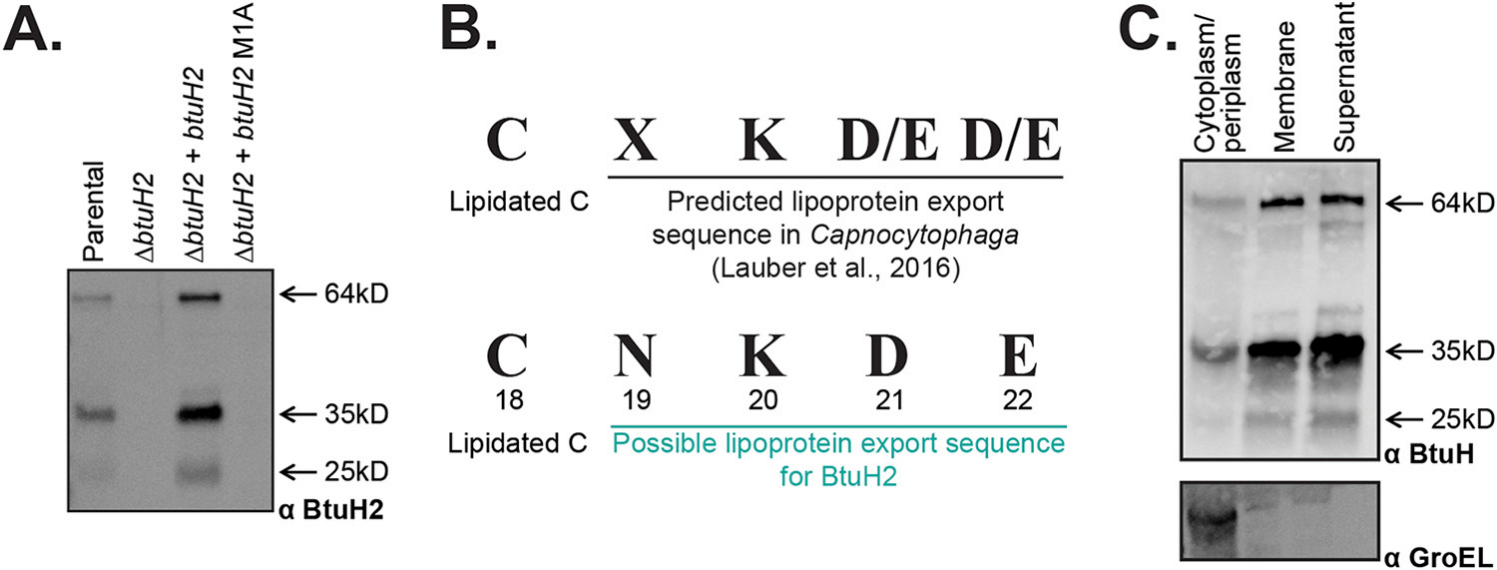
BtuH2 encodes a predicted lipoprotein export sequence and is membrane-associated. (A) Immunoblot analysis of the B. thetaiotaomicron parental strain, using a polyclonal antibody raised against BtuH2-His_10_. Bands consistent with the expected size of the full-length mature protein (64 kD), as well as the two additional fragments, are absent in the Δ*btuH2* strain, present in the complemented strain, and absent if the initial methionine is mutated to alanine (M1A). (B) The N-terminal amino acids from BtuH2, C18 to E22, match the predicted lipoprotein export sequence for *Bacteroides* spp. (19). (C) Cell fractionation studies reveal that BtuH2 is enriched in the membrane and supernatant fractions. A cytoplasmic control (GroEL) is shown.

To further explore the role of BtuH2 in cobamide transport, we raised a polyclonal antibody against recombinant BtuH2-His_10_ purified from E. coli. Western blots of B. thetaiotaomicron whole-cell lysates showed the full-length BtuH2 at the expected size of 64 kDa and additional bands at 35 kDa and 25 kDa (Fig. 3A). These bands were absent from whole-cell lysates from a Δ*btuH2* mutant strain, indicating that they are specific to this protein (Fig. 3A). Replacing the initiating methionine with alanine eliminates all three bands, suggesting that these fragments are likely products of protein cleavage or degradation rather than multiple translational start sites (Fig. 3A).

BtuH2 is predicted to contain a lipoprotein export sequence at its N terminus (Fig. 3B) (19). Indeed, cell fractionation studies localize BtuH2 (64-, 35-, and 25-kDa fragments) to both membrane and supernatant fractions, with minimal protein detected in the cytoplasm (Fig. 3C). BtuH2 cleavage does not require the other membrane-associated B_12_ transport proteins BtuG2 or BtuB2 (see Fig. S2A) or any proteins encoded in the three vitamin B_12_ transport loci (see Fig. S2B). Further, immunoprecipitation of BtuB2 identifies BtuG2 as a stable interacting partner as expected (13), but does not enrich for BtuH2 (see Fig. S2C). Similarly, immunoprecipitation of BtuH2 does not robustly enrich for BtuG2, suggesting that BtuH2 function may not require strong interactions with BtuB2 or BtuG2 (see Fig. S2D).

10.1128/mBio.02845-21.3 FIG S2 Conditions that retain BtuB2-BtuG2 interaction do not provide evidence of direct interaction between BtuH2 and these cobamide transport proteins. (A) BtuH2 cleavage does not require BtuG2 or BtuB2. Immunoblots show whole-cell lysates of the B. thetaiotaomicron parental strain and isogenic Δ*btuB2*, Δ*btuG2*, and Δ*btuH2* mutants probed with polyclonal antibodies against BtuG2 and BtuH2. (B) BtuH2 cleavage does not require any of the other proteins encoded in the three vitamin B_12_ transport loci. The immunoblot shows whole-cell lysates of the B. thetaiotaomicron parental strain, Δ*btuH2* mutant, complement, and a strain carrying the same *btuH2* complementation construct in which all three vitamin B_12_ transport loci have been deleted, probed using polyclonal antibodies against BtuH2. (C) Immunoprecipitation of BtuB2-FLAG-HA using anti-HA resin followed by immunoblotting against HA to detect BtuB2, or using polyclonal antibodies to detect BtuG2 and BtuH2. (D) Immunoprecipitation of BtuH2, using anti-BtuH2 polyclonal antibodies conjugated to protein A-Dynabeads, followed by immunoblotting with antibodies against BtuH2 and BtuG2. Download FIG S2, TIF file, 3.6 MB. Copyright © 2022 Putnam et al. 2022 Putnam et al. https://creativecommons.org/licenses/by/4.0/ This content is distributed under the terms of the Creative Commons Attribution 4.0 International license.

### The crystal structure of BtuH2 reveals a C-terminal domain that binds vitamin B_12_.

**FIG 4:**
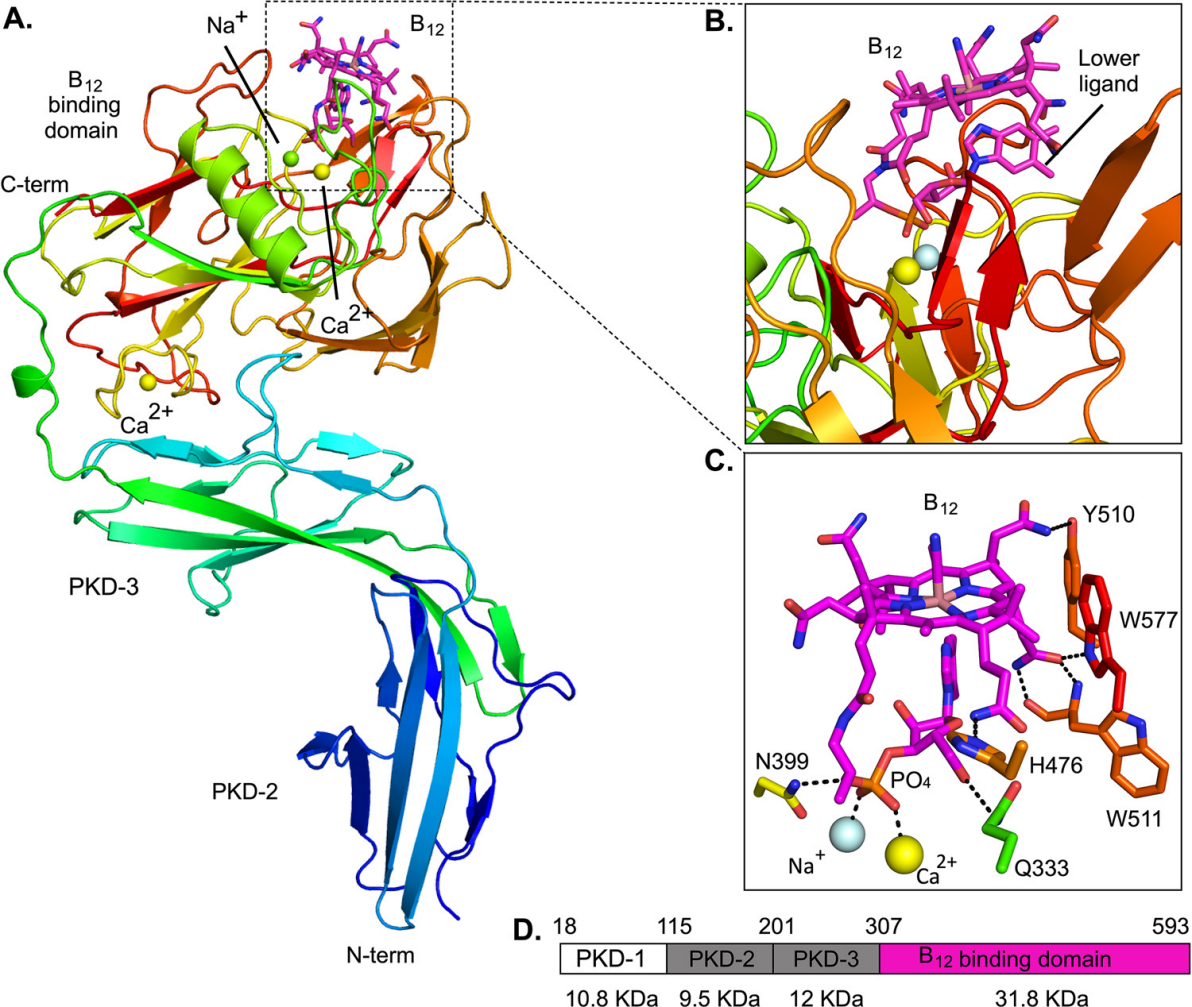
Crystal structure of BtuH2 in complex with B_12_ shows a C-terminal ligand-binding domain and N-terminal Ig-like PKD domains. (A) Cartoon model of BtuH2 in rainbow coloring ramped from blue at the N terminus to red at the C terminus. Calcium (yellow spheres) and sodium (pale cyan sphere) atoms and vitamin B_12_ (magenta sticks) are shown. (B) Close-up of the B_12_ binding site. Note that the lower ligand is pointing toward the protein. (C) Representation of the polar interactions between BtuH2 and B_12_ and of ionic bonds between calcium and sodium with the B_12_ phosphate group. (D) Schematic representation of the BtuH2 mature peptide and its structural domains. Top numbers represent the amino acids. The approximate size of each fragment is indicated.

Attempts to obtain well-diffracting crystals of full-length BtuH2 proved to be unsuccessful. As mentioned above, sequence analysis predicts three PKD domains in the N-terminal half of the sequence. We next constructed and expressed a version of BtuH2 excluding the most N-terminal PKD domain. The truncated protein includes residues 103 to 593 with a C-terminal His_6_ tag. This shorter version produced well-diffracting crystals, allowing us to solve the structure of cyanocobalamin-bound BtuH2_(103-593)_-His_6_ via cobalt-based single-wavelength anomalous diffraction (Co-SAD; see Text S1 and Table S2 in the supplemental material). The solved structure has three domains; two Ig-like N-terminal PKD domains (Ig-like 2 and 3 in the full-length protein) and a globular C-terminal domain (Fig. 4A and D). The predicted most N-terminal PKD domain is not present in our construct but likely has a similar Ig-like fold. The N-terminal region shows high flexibility and is only well-structured in one of the two chains present in the asymmetric unit (chain A).

10.1128/mBio.02845-21.9 TABLE S2 Crystallographic tables. Download Table S2, DOCX file, 0.02 MB. Copyright © 2022 Putnam et al. 2022 Putnam et al. https://creativecommons.org/licenses/by/4.0/ This content is distributed under the terms of the Creative Commons Attribution 4.0 International license.

As expected from the successful structure solution via Co-SAD, clear electron density is visible for a vitamin B_12_ molecule bound in a cleft on the C-terminal domain (Fig. 4B), allowing unambiguous placement of the ligand. There are two main regions of interaction between B_12_ and BtuH2: the tetrapyrrole ring and the nucleotide loop. Residues Tyr510, Trp577, Trp511, and His476 form hydrogen bonds with the amide functional group of the substituents in C-7, C-8, and C-13 of the tetrapyrrole ring, whereas Asn399 and Gln333 mediate polar interactions with the phosphate and the -CH_2_OH functional group of the ribose, respectively (Fig. 4C). The interaction between the dimethylbenzimidazole (DMB) lower ligand and BtuH2 is likely weak, consisting only of van der Waals interactions with Leu578 and Phe401 (see Fig. S3). There is no interaction between BtuH2 and the B_12_ upper ligand. The nucleotide loop interactions, including those mediated by Asn399 and Gln333, and the lack of strong interactions with the lower ligand, suggest that BtuH2 primarily interacts with vitamin B_12_ via its tetrapyrrole ring and nucleotide loop, which are conserved across cobamides.

10.1128/mBio.02845-21.4 FIG S3 BtuH2 residues implicated in B_12_ and metal binding colored by ConSurf conservation score. (A) Close-up view of the residues directly interacting with vitamin B_12_. The metal atoms, Ca^2+^ and Na^+^, interact with the phosphate group of B_12_. Black dash lines represent hydrogen and metal bonds. (B) Cartoon representation showing the hydrophobic interactions. (C) The metal atoms located in the B_12_ binding pocket interact with two water molecules (red spheres) and highly conserved amino acids. For clarity, the phosphate group and the rest of the B_12_ structure are not shown. (D) Cartoon showing the side chain of the conserved residues and the carbonyl group of the peptide bond from the nonconserved leucine interacting with the calcium located close to the domain interface. Download FIG S3, TIF file, 2.7 MB. Copyright © 2022 Putnam et al. 2022 Putnam et al. https://creativecommons.org/licenses/by/4.0/ This content is distributed under the terms of the Creative Commons Attribution 4.0 International license.

A comparison of the B_12_ binding domain against all the structures deposited in the PDB using the DALI server (20) shows low structural homology with any other known structure (see Table S2). To detect potential functionally and structurally important residues, we carried out an evolutionary analysis using the ConSurf web server (21, 22). The analysis showed that the residues located on the protein surface are poorly conserved except for two regions: the B_12_ binding pocket in the vicinity of the phosphate group, and the interface region between the globular C-terminal domain and the Ig-like domain 3 close to the linker (see Fig. S5). Beside these two surface regions, the core amino acids of the globular domain are conserved (see Fig. S5). This could explain the modest structural similarity from the DALI analysis with structures that have a similar globular domain, such as the periplasmic alginate lyase AlyQ, and suggests that the structural similarities are not related to the B_12_ binding function of BtuH.

10.1128/mBio.02845-21.6 FIG S5 Sequence conservation of BtuH2 colored by ConSurf conservation score. The conservation score was calculated from an alignment of 150 orthologs. (A) Surface view of BtuH2 in several orientations. Open dashed red squares highlight the conserved surface regions. The close-up view shows the conserved bottom region of the B_12_ binding pocket. (B) Cartoon model showing the conserved regions around the calcium and sodium atoms and the core of the globular domain. Download FIG S5, TIF file, 1.6 MB. Copyright © 2022 Putnam et al. 2022 Putnam et al. https://creativecommons.org/licenses/by/4.0/ This content is distributed under the terms of the Creative Commons Attribution 4.0 International license.

The crystallographic data also showed three round blobs of density (strong peaks of 14, 24, and 25σ) that do not belong to B_12_ or the protein (see Fig. S4). After inspection of the residues surrounding the electron density, *in silico* analyses of the geometry and coordination using METALizer (23) and the CheckMyMetal (24) server, and the analysis of anomalous signal peaks we modeled these as one sodium and two calcium atoms (Fig. 4A; see also Fig. S4). The conservation and the network of interactions bridged by the metals with the nucleotide loop group suggest an important role of this region for BtuH2 function (see Fig. S3). The calcium ion located at the interface between the Ig-like domain 3 and the B_12_ binding domain is coordinated only with amino acids from the globular domain. The ConSurf analysis shows that all the amino acids that interact with this calcium via their side chains are highly conserved (see Fig. S3), suggesting an important and most likely structural role for this calcium ion.

10.1128/mBio.02845-21.5 FIG S4 Metal analysis. (A to E) Map coefficients of the ions interacting with vitamin B_12_. The high-resolution data were collected at 12.8 KeV, at which energy the anomalous signal of sodium is small (0.05 e^–^ compared to 0.56 e^–^ for calcium and 1.7 e^–^ for cobalt). Hence, the two density peaks that also had a strong anomalous signal (∼10 σ; compare with ∼28 σ for Co) were modeled as calcium, and the peak density lacking anomalous signal was modeled as sodium. We also modeled the blobs both as calcium or sodium, but the presence of clear difference density supported the original assignment of one calcium and one sodium. The calcium and sodium atoms close to B_12_ molecule are located 3.7 Å from each other, and coordinate with highly conserved protein residues, two water molecules, and with the phosphate of the nucleotide loop of B_12_. The figure provides views after various refinements showing 2Fo-Fc electron density contoured at 1.5 σ (blue), as well as Fo-Fc difference maps, with negative electron density in red and positive in green (contoured at 3 σ) and an anomalous difference map contoured at 4.4 σ in pink. The yellow sphere represents calcium and the cyan sphere represents sodium. A partial view of B_12_ is shown in magenta. All maps were carved within 1.6 Å from the metals and B_12_. (A) Two calcium atoms are modeled. (B) One calcium and one sodium are modeled. (C) Two sodium atoms are modeled. (D) Atoms were swapped compared to panel B. (E) Same as panel D but showing the anomalous difference map. Notice the discrepancies (red and green mesh) between the measured electron density of the crystal (blue mesh) and the electron density explained by the modeled atoms. Only with the ion combination shown in panel D the measured electron density agrees with the model. In addition, the anomalous data support the type and the chosen disposition of the atoms modeled from panel D. At the particular wavelength used to collect data, calcium but not sodium behaves as an anomalous scatter. The anomalous difference map (pink mesh in panel E) shows an anomalous signal only for one of the electron density blobs, the same one whose measured electron density is explained only by a calcium atom and not by a sodium atom. (F, G, and H) Metal analysis for selected metals from chain A. (F) Metal analysis using CheckMyMetal. (G) Metal analysis using METALizer. For both panels F and G, atoms F1 and D1 form the dinuclear metal center. nVECSUM, summation of ligand vectors, weighted by bond valence values and normalized by overall valence. gRMSD(°), root mean square deviation (RMSD) of observed geometry angles (L-M-L angles) compared to ideal geometry, in degrees. Score, the total score is the weighted sum of the geometry RMDS, the number of free sites, and the overlap penalty. (H) Distances between metals and their coordination atoms (BtuH residues, B_12_, or water molecules). Download FIG S4, TIF file, 1.4 MB. Copyright © 2022 Putnam et al. 2022 Putnam et al. https://creativecommons.org/licenses/by/4.0/ This content is distributed under the terms of the Creative Commons Attribution 4.0 International license.

### *btuH* homologs are widespread among gut *Bacteroidetes*.

**FIG 5:**
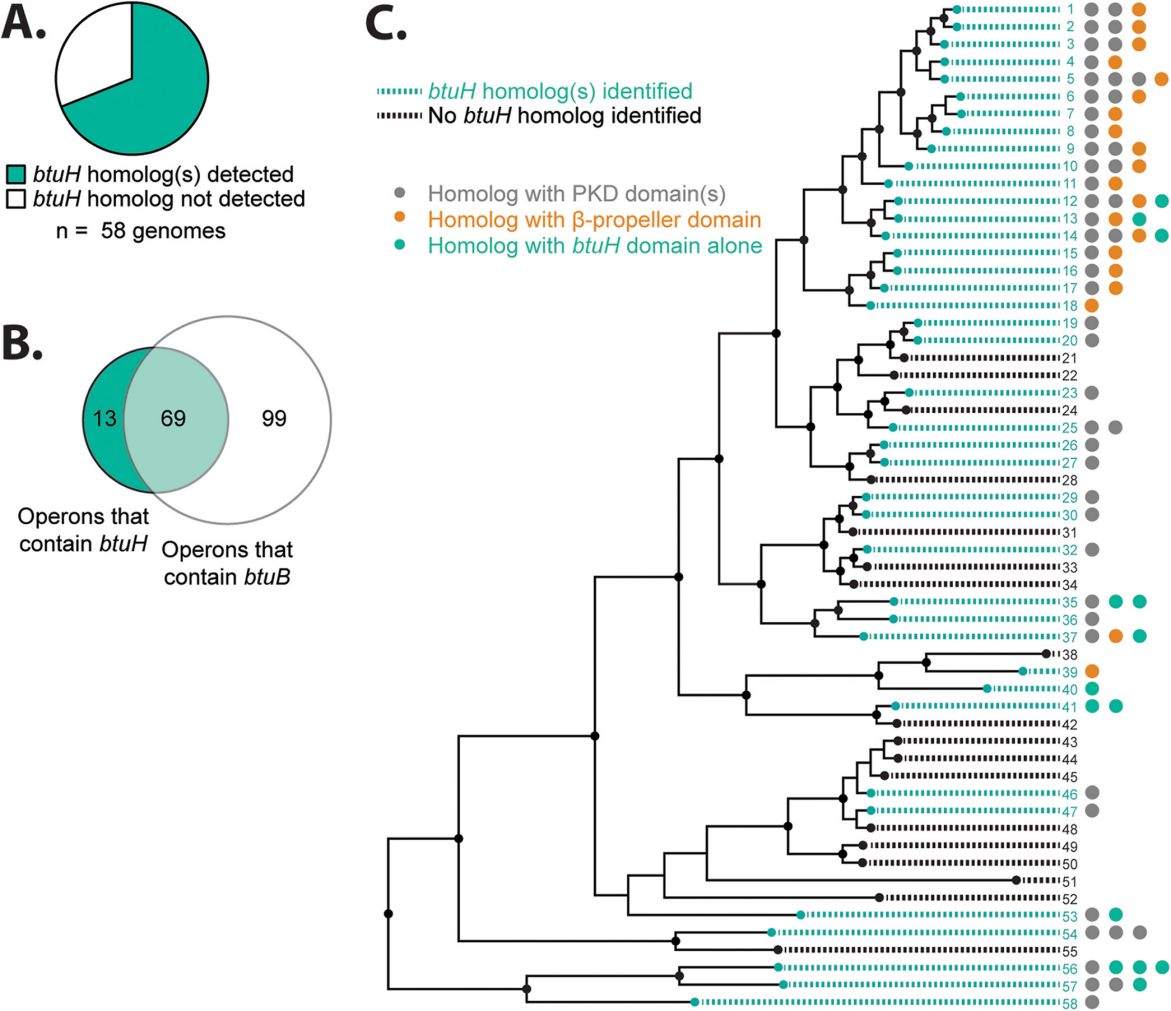
*btuH* homologs are widespread among gut *Bacteroidetes. (*A*)* Presence of one or more *btuH* homologs in gut *Bacteroidetes* genomes (*n* = 58 genomes). (B) Gut *Bacteroides* operons containing *btuH* homologs, *btuB* homologs, or both (*n* = 181 operons). (C) Distribution of *btuH* homologs across the gut *Bacteroidetes* phylogeny. Strains that encode at least one *btuH* homolog are shown in teal and strains with no identified *btuH* homologs are shown in black. Numbers along the right side correspond to the species number in Table S3A. Dots along the right side indicate the number of homologs, as well as the associated domains (teal for no additional domains, orange for β-propeller, and gray for PKD domain).

We next examined the phylogenetic distribution of *btuH* homologs across a panel of 313 genome-sequenced human gut bacterial species whose cobamide transport and biosynthetic capacities have been previously defined (12). Approximately two-thirds of gut *Bacteroidetes* in this panel encode predicted BtuH homolog(s) (Fig. 5A; see also Table S3A), and approximately 40% of the predicted vitamin B_12_ transport loci among gut *Bacteroidetes* include a *btuH* homolog (Fig. 5B). More than 80% of the *btuH* homologs identified from the 313 species are predicted to belong to an operon that also includes *btuB* (Fig. 5B). This consistent association of *btuH* homologs with genes encoding known cobamide transport machinery suggests that BtuH is involved in cobamide acquisition in species beyond B. thetaiotaomicron.

10.1128/mBio.02845-21.10 TABLE S3 BtuH homologs and strains and primers. (A) BtuH homologs identified from dataset of 313 genome-sequenced gut species. (B) Strains and primers used in this study. Download Table S3, XLSX file, 0.03 MB. Copyright © 2022 Putnam et al. 2022 Putnam et al. https://creativecommons.org/licenses/by/4.0/ This content is distributed under the terms of the Creative Commons Attribution 4.0 International license.

Notably, unlike the cobamide transport components conserved across Gram-negative bacteria (*btuBFCD*) or across gut *Bacteroidetes* (*btuG*), *btuH* homologs are heterogeneously distributed across the phylogenetic tree of gut *Bacteroidetes* (Fig. 5C; see also Table S3A). Moreover, species phylogeny does not predict the number of *btuH* homologs or which *btuH* alleles (i.e., *btuH* genes encoding N-terminal PKD, β-propeller, or other domains) are encoded in each genome (Fig. 5C; see also Table S3A). These observations are consistent with horizontal gene transfer of *btuH*; this has been previously suggested for *btuB* (12).

### The abundance of *btuH* homologs in gut *Bacteroidetes* genomes is inversely correlated with predicted cobamide biosynthetic ability.

**FIG 6:**
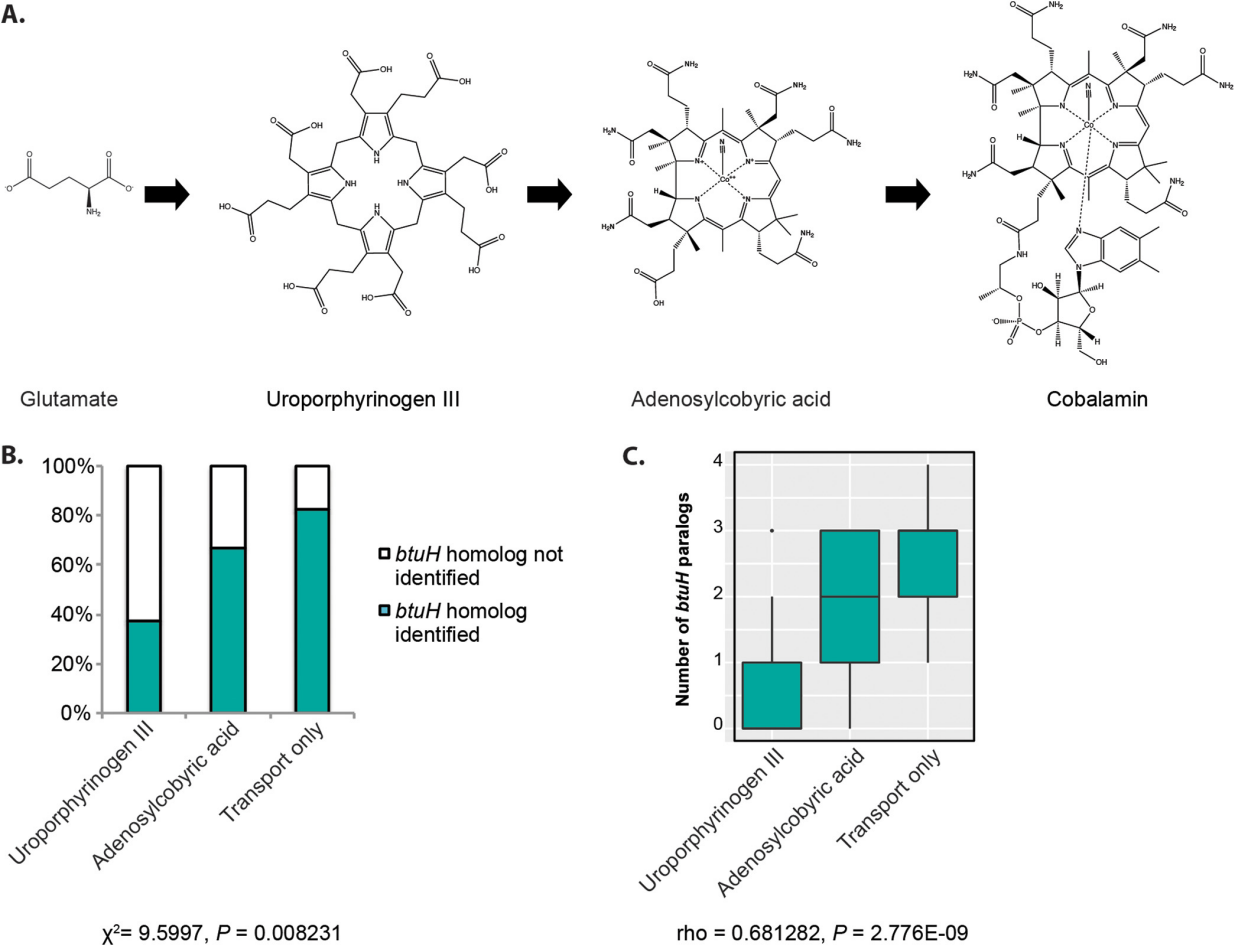
Gut *Bacteroidetes* that encode limited vitamin B_12_ biosynthetic ability are enriched in *btuH* homologs. (A) A simplified schematic of the vitamin B_12_ biosynthesis pathway. Microbes that encode vitamin B_12_-dependent enzymes can use glutamate for *de novo* biosynthesis of the vitamin, salvage early-stage precursors such as uroporphyrinogen III, salvage late-stage precursors such as adenosylcobyric acid, or rely on completed cobamides that are acquired by transport. Among the gut *Bacteroidetes*, only the latter three categories are reported (12). (B) Gut *Bacteroidetes* with limited cobamide biosynthetic capacity are significantly more likely to encode *btuH*. The percentage of strains that encode one or more *btuH* homolog are shown in teal, while those without *btuH* homologs are shown in white. Significance was assessed by χ^2^ test (*n* = 58 genomes, χ^2^ = 9.5997, *P* = 0.008). (C) The number of *btuH* gene paralogs is significantly inversely associated with cobamide biosynthetic capacity. The number of *btuH* paralogs identified in strains in each group is shown on the *y* axis (Spearman correlation, *n* = 58 genomes, rho = 0.68128, *P* = 2.776E-09). Box plots indicate the median with first and third quartiles, and whiskers indicate the highest and lowest values not more than 1.5 times the interquartile range. Data points outside this range are shown as individual dots.

Since *btuH* homologs appear to be gained and lost repeatedly across the *Bacteroidetes* phylogeny, we next asked which features are associated with the presence or abundance of these genes. Cobamide biosynthetic and remodeling capacity varies widely across gut *Bacteroidetes* (Fig. 6A) (6, 12). To investigate whether these variable capacities are associated with the observed heterogeneity in *btuH* gene abundance, we first analyzed 58 *Bacteroidetes* genomes whose cobamide biosynthetic capacities were previously classified (12). Notably, *Bacteroidetes* species that encode a more complete cobamide biosynthetic pathway are significantly less likely to encode *btuH* (Fig. 6B, χ^2^ test, χ^2^ = 9.5997, *P* = 0.008). The number of *btuH* paralogs in each genome follows a similar pattern (Fig. 6C, Spearman correlation, rho = 0.68128, *P* = 2.776E–09). In a separate data set of 89 animal gut *Bacteroidetes* genomes whose cobamide biosynthesis capacity was classified using a different approach (6), *btuH* gene copy number is similarly inversely correlated with B_12_ biosynthetic capacity (see Fig. S6, Spearman correlation, rho = 0.47581, *P* = 2.448E–06). Together, these analyses suggest that, while *btuH* homologs are frequently gained and lost across the phylogeny, these proteins are preferentially maintained in species with limited cobamide biosynthetic capacity.

10.1128/mBio.02845-21.7 FIG S6 Analysis of the association between *btuH* homolog count and vitamin B_12_ biosynthetic ability in an independent dataset. From the dataset reported in (6), only the *Bacteroidetes* with known associations with animal gastrointestinal tracts are shown. Within this subset of the *Bacteroidetes*, all of the strains encode transport-related genes but only some of the strains encode the machinery to salvage the cobamide precursor, cobinamide. The average number of *btuH* paralogs per strain identified in each group is shown on the *y* axis (Spearman correlation, *n* = 89 genomes, rho = 0.47581, *P* = 2.448E–06). Box plots indicate the median with first and third quartiles, and whiskers indicate the highest and lowest values not more than 1.5 times the interquartile range. Data points outside this range are shown as individual dots. Download FIG S6, TIF file, 1.9 MB. Copyright © 2022 Putnam et al. 2022 Putnam et al. https://creativecommons.org/licenses/by/4.0/ This content is distributed under the terms of the Creative Commons Attribution 4.0 International license.

## DISCUSSION

In the present study, we used an affinity-based probe to identify vitamin B_12_-binding proteins in B. thetaiotaomicron. In addition to vitamin B_12_ acquisition proteins, B. thetaiotaomicron encodes five known vitamin B_12_-binding proteins or protein complexes, including MetH (B_12_-dependent methionine synthase; BT_0180), Mta (methylamine/trimethylamine methyltransferase corrinoid proteins; BT_0340, BT_0342, BT_0343), HpnR*/*Hyp (B_12_-binding domain/radical SAM domain protein; BT_0640), MutAB (methylmalonyl-CoA mutase; BT_2090 and BT_2091), and NrdJ (B_12_-dependent ribonucleotide reductase; BT_2145) (12, 16, 25–27). Of these, four (MetH, HpnR/Hyp, NrdJ, and MutAB) bound to B_12_-ABP in one or both replicates of the screen (see Table S1). In addition, known components of the B_12_ transport machinery (homologs of BtuB, BtuF, and BtuG) were significantly enriched in one or both replicates of the screen. These results suggest that B_12_-ABP can identify genuine vitamin B_12_-binding proteins but may under sample the total repertoire of such proteins in B. thetaiotaomicron.

The presence of known vitamin B_12_-binding proteins in the screen suggests that other candidates not previously identified as B_12_-dependent also interact with this cofactor. For example, BT_1491 was identified as a B_12_-binding protein. Since BT_1491 is hypothetical protein in a B_12_-transport associated operon, this suggests that there may be additional novel B_12_-acquisition proteins. Outside of the B_12_-transport operons, the screen identified multiple proteins with similar predicted functions. For example, BT_2079 is a predicted peptidyl-prolyl *cis-trans* isomerase that bound B_12_-ABP in both replicates of the screen (see Table S1). Three other peptidyl-prolyl isomerases (BT_2976, BT_3841, and BT_4371) bound to B_12_-ABP in one replicate of this screen (see Table S1). Vitamin B_12_ (specifically adenosylcobalamin) can function as a cofactor for other isomerases (17) but, to our knowledge, this is the first suggestion of cobamides interacting specifically with peptidyl-prolyl isomerases.

In this study, we characterized BtuH2, a representative of a previously uncharacterized protein family identified in the B_12_-ABP screen. These proteins are absent from E. coli and present in only a subset of *Bacteroidetes* cobamide transport loci; however, *btuH2* is required for efficient vitamin transport by the cobamide transport system encoded by neighboring genes. It is not known how vitamin B_12_ capture and transport are coordinated between components of the cobamide transport machinery that are shared across Gram-negative phyla (e.g., BtuBFCD), components encoded by genes present in all *Bacteroidetes* cobamide transport loci but absent from E. coli (e.g., BtuG), and components present in some *Bacteroidetes* transport loci but not others (e.g., BtuH). Our data indicate that BtuH2 is a membrane-associated lipoprotein that binds vitamin B_12_ directly, suggesting a role for BtuH2 in cobamide capture at the cell surface. Previous work demonstrated that three BtuB proteins are the only outer membrane transporters for cobamides (12), so any cobamides captured by BtuH likely cross the outer membrane via one or more of these BtuB transporters. We observed no evidence of enrichment of BtuH2 in a BtuB2 immunoprecipitation, or enrichment of BtuG2 in a BtuH2 immunoprecipitation, suggesting that these surface-exposed outer membrane proteins do not form a stable complex under the conditions tested. Transient protein-protein interactions that were not captured under our study conditions, lack of direct interaction, or unidentified intermediate proteins may explain these results. In addition, vitamin B_12_ transport proteins encoded in different loci may interact with each other. One of more of these proposed mechanisms could also be involved in releasing B_12_ from BtuH2 so that the B_12_ can be transported into the cell.

The crystal structure of BtuH2 indicates that this protein binds B_12_ via a domain that is not shared with previously characterized B_12_-binding proteins or any other previously characterized proteins. Notably, the protein captures B_12_ without strong interactions to the B_12_ lower ligand. Since the lower ligand varies among different cobamides, this feature could potentially allow BtuH-family proteins to contribute to cobamide capture by different transport systems that preferentially capture distinct cobamides from the gut environment.

Homologs of *btuH* are widespread but not ubiquitous across gut *Bacteroidetes*. From a set of 313 genome-sequenced gut commensals, approximately two-thirds of the *Bacteroidetes* encode one or more predicted *btuH* homologs. Interestingly, three *Firmicutes* species encode predicted *btuH* homologs. While less is known about cobamide acquisition in the *Firmicutes*, one of these *btuH* homologs, EUBSIR_01285 in Eubacterium siraeum DSM 15702, is encoded near the gene for a predicted ECF-like cobamide transporter. Within the gut *Bacteroidetes*, *btuH* genes are heterogeneously distributed across the phylogeny (Fig. 5C), suggesting that these genes are frequently mobilized by horizontal gene transfer. The presence of predicted *btuH* homologs in another phylum is consistent with this possibility.

Genomic analysis further reveals that gut *Bacteroidetes* vary in the number of predicted *btuH* copies encoded, as well as the configurations of associated domains (Fig. 5C). Why some but not all gut *Bacteroidetes* encode *btuH*, and why different numbers of paralogs are maintained in different species, is not known. The inverse correlation between biosynthetic capability and *btuH* (Fig. 6; see also Fig. S6) suggests that *btuH* may be particularly beneficial for fitness in gut *Bacteroidetes* that rely on capturing mature cobamides from the environment. Together, these studies suggest that gut *Bacteroidetes* assemble a heterogeneous set of cobamide transport machineries comprised of distinct, functional components. Future studies will establish the connections between vitamin biosynthetic capacity and these specialized mechanisms for vitamin scavenging.

## MATERIALS AND METHODS

### Bacterial strains and growth conditions.

Bacterial strains and primers used in this study are listed in Table S3B. B. thetaiotaomicron was grown in a flexible anaerobic chamber (Coy Laboratory Products), filled with 70% N_2_, 20% CO_2_, and 10% H_2_. The culture medium for B. thetaiotaomicron was a defined minimal medium (28), supplemented with 500 μM d,l-methionine instead of vitamin B_12_, unless otherwise noted. E. coli was grown aerobically at 37°C at 225 rpm in Luria-Bertani broth. Antibiotics were supplemented as needed at the following concentrations: ampicillin, 100 μg/mL; gentamicin, 200 μg/mL; erythromycin, 25 μg/mL; tetracycline, 2 μg/mL; and 5-fluoro-2′-deoxyuridine (FUdR), 200 μg/mL.

### B_12_-ABP proteomics profiling.

B. thetaiotaomicron *tdk* was grown in minimal medium supplemented with methionine. Overnight cultures were washed, back diluted to a starting optical density at 600 nm (OD_600_) of ∼0.0001 and supplemented with either vitamin B_12_ (cyanocobalamin; Sigma, V6629) or B_12_-ABP (14) to a final concentration of 10 nM. Log-phase cultures were harvested by centrifugation, washed with sterile phosphate-buffered saline (pH 7.2; PBS), and resuspended in PBS. Cells were then irradiated for 10 min (UVP Black-Ray XX-15BLB lamp, two bulbs, 15 W, 365 nm, 12 cm from lamp to sample) to cross-link the probe to adjacent proteins. After irradiation, the cells were pelleted and frozen.

Probe-labeled cell pellets were thawed and lysed via sonication. Protein concentration for each sample was determined with a bicinchoninic acid assay, and samples were normalized to a concentration of 1 mg/mL in 500 μL of PBS. Click chemistry, streptavidin-based enrichment of probe-labeled proteins, and sample preparation for liquid chromatography-tandem mass spectrometry analysis were performed as described previously (29), except the samples in data set B were not reduced or alkylated. All proteomics samples were analyzed using a Velos Orbitrap MS apparatus, as previously described (30).

MaxQuant (31) was used for peptide-spectrum matching and calculation of protein LFQ intensities (32). Raw liquid chromatography-mass spectrometry files for each data set were loaded into MaxQuant version 1.6.3.4. Variable modifications included methionine oxidation and N-terminal acetylation. Carbamidomethylation was selected as a fixed modification for the data set that was reduced and alkylated. Trypsin was selected as the digestive enzyme. Samples for each data set were matched between runs and all other options, including LFQ parameters, were set to their default parameters. The Bacteroides thetaiotaomicron (Table S1) FASTA file used in the MaxQuant search was obtained from UniProt (33) and contained 4,782 protein sequences. The resulting LFQ intensities in the proteinGroups.txt file from MaxQuant were used for comparing protein enrichment between samples. Potential contaminants and proteins identified by fewer than three peptides were removed prior to further analysis.

For each sample comparison, proteins were selected for further analysis if abundances were observed in all four replicates in at least one of the compared samples. Abundances were then log_2_ transformed. Any missing values were imputed by first filling in missing values with the minimum observed abundance across samples. The average of the observed and populated abundances was then used to create a normal distribution with a standard deviation equal to the average standard deviation of abundances across compared samples. Randomly selected values from the distribution were then selected as effective abundances for each protein where any missing values in compared samples were present. Protein abundances were then statistically compared using a right-side, two-sample *t* test to detect enriched proteins. The Benjamini-Hochberg false discovery rate (FDR) correction method (34) was performed on *P* values to correct for multiple-hypothesis testing. Proteins with FDR-adjusted *P* values of <0.05 were determined to be significantly enriched B_12_-ABP targets. In addition, fold changes were calculated as the difference in average replicate log_2_ protein abundances between samples.

### Genetic techniques.

Genetic techniques were performed as previously described (13). Plasmids were constructed using standard molecular biology techniques. Plasmids were introduced into E. coli via electroporation and into B. thetaiotaomicron via conjugation from E. coli S17-1 λ*pir*. Complementation and barcoding constructs in the pNBU2 vector backbone were screened via PCR for integration at either the att1 or att2 site in B. thetaiotaomicron. Deletion and allelic exchange constructs in the pExchange-*tdk* plasmid (35) contained ∼1-kb flanking regions for integration at the native locus in B. thetaiotaomicron. FudR was used for counterselection for loss of the plasmid vector.

### Surface plasmon resonance.

BtuH2-His_10_ was purified from E. coli BL21 Rosetta(DE3) as described above. Protein used for SPR analysis was additionally purified by size exclusion chromatography on an S200 column. SPR was performed using previously described methods (13). The results are reported as an average of two independent runs on independent protein purifications.

### Growth curves.

B. thetaiotaomicron parental, Δ*btuH2*, and complemented strains were grown in minimal medium with methionine. Overnight cultures were washed three times with minimal medium lacking both methionine and vitamin B_12_. Cultures were then diluted into fresh minimal medium without methionine, supplemented with the indicated concentrations of vitamin B_12_, at a starting OD600 of 0.001. Cultures were grown anaerobically at 37°C with constant mixing, and OD_600_ measurements were collected hourly on a BioTek Eon plate reader.

### Gnotobiotic studies.

Animal experiments were performed with protocols approved by the Yale University Institutional Animal Care and Use Committee. Germfree Swiss-Webster mice were individually caged and housed in flexible plastic gnotobiotic isolators. Mice were fed a standard mouse chow diet *ad libitum* (5K67 LabDiet; Purina, St. Louis, MO). Barcoded versions of B. thetaiotaomicron parental, Δ*btuH2* mutant, and complemented strains were separately grown overnight in TYG. Cultures were frozen in 20% (vol/vol) glycerol in multiple identical aliquots. One aliquot of each strain was thawed and plated to determine the CFU/ml. Immediately prior to gavaging mice, cultures were thawed and normalized to 5 × 10^8^ CFU/mL in sterile PBS. These normalized cultures were then combined at a 1:8:1 ratio (parental:Δ*btuH2*:Δ*btuH2*+*btuH2*). Five germfree Swiss-Webster mice, 9 weeks old, were each gavaged with 200 μL of this mixture. Fecal pellets were collected on days 1, 2, 3, 6, 9, 12, and 15 for analysis by qPCR of the individual barcodes encoded in each strain.

### qPCR.

From mouse fecal pellets, DNA was extracted as previously described using phenol-chloroform extraction, precipitation in isopropanol, and further purification on a Qiagen PCR cleanup column (36). Purified DNA was then diluted 1:100 in nuclease-free water and used as the template for qPCR. Standard curves were generated from B. thetaiotaomicron *att1*::pNBU2_tet strains containing each of the three barcodes used. Cells for standard curve generation were grown overnight in TYG, pelleted, DNA extracted as described above, and quantified. Standard curves were generated with genomic DNA ranging from 100 to 0.0001 ng. qPCR was done using KAPA SYBR FAST qPCR Mix (KAPA KK4600). Absolute and relative abundance of each barcoded strain was calculated based on standard curves.

### qRT-PCR.

The B. thetaiotaomicron Δ*tdk*, parental, Δ*btuH2*, and Δ*btuH2*+*btuH2* strains were grown in minimal media with methionine and 0, 1, or 10 nM vitamin B_12_. Cells were harvested at mid-log phase (OD_600_ of 0.30 to 0.50), and RNA was extracted using an RNeasy kit (Qiagen). RNA samples were treated with Turbo DNase (Thermo Fisher) and cleaned a second time on RNeasy columns. cDNA was made using SuperScript III reverse transcriptase (Invitrogen) and cleaned using QIAquick columns (Qiagen). Single-stranded cDNA was quantified using a Qubit prior to running qPCRs, as described above. Data were analyzed using the ΔΔ*C_T_* method (37), in which 16S gene expression was the housekeeping gene and 0 nM vitamin B_12_ was the untreated condition.

### Transcriptional reporter assay.

Transcriptional reporter strains were constructed by fusing predicted B_12_-responsive riboswitches to the reporter gene *nanoluc* in the vector pNBU2_Erm. Constructs were conjugated into the B. thetaiotaomicron Δ*tdk* strain, and insertion into the *att1* locus was confirmed by colony PCR. Strains were grown in minimal medium containing methionine but no B_12_ and harvested at mid-log phase (OD_600_ 0.30 to 0.50). Cells were washed and resuspended in PBS and then lysed using BugBuster (Novagen). Equal volumes of cell lysate and Nano-Glo luciferase assay reagent (Promega) were incubated for 3 min at room temperature, and the luciferase activity was measured with integration time of 1 s at a gain setting of 100 using a BioTek Synergy H1 plate reader.

### Immunoblotting.

BtuH2 was detected in B. thetaiotaomicron whole-cell lysates using a custom rabbit polyclonal antibody (Cocalico Biologicals), raised against BtuH2-His_10_. BtuG2 was detected using a previously reported rabbit polyclonal antibody (13) and BtuB2-FLAG-HA was detected using a rabbit polyclonal anti-HA antibody (Sigma, H6908). Whole-cell lysates were prepared by boiling cells in Laemmli buffer prior to SDS-PAGE.

### Cell fractionation.

B. thetaiotaomicron strains were grown in minimal medium to log phase. Cells were harvested and separated from the supernatant by centrifugation. Cells were then resuspended in breakage buffer (50 mM Tris [pH 7.4], 5 mM EDTA, 2 mM phenylmethylsulfonyl fluoride, 10% glycerol) and lysed by sonication. The lysate and supernatant were filtered (0.2-μm pore size) to remove any whole cells before centrifugation at 100,000 × *g* at 4°C for 1 h. The pelleted membrane fraction was washed and resuspended in breakage buffer. The soluble and membrane fractions were then centrifuged a second time at 100,000 × *g* at 4°C for 1 h. Membrane pellets were resuspended in breakage buffer. The soluble fraction of the cells, and the supernatant fraction were concentrated using Amicon centrifugation, such that all three fractions were equal volume prior to analysis by immunoblotting. GroEL was assessed by immunoblot as a control for cytoplasmic proteins (rabbit anti-GroEL; Sigma, G6532).

### Identification of *btuH* homologs.

To identify *btuH* homologs for comparative genomic analysis, we used a previously published data set of 313 genome sequenced gut species (12). Within this data set, we individually searched by BLASTX for homologs of *btuH1* (*bt1488*), *btuG1* (*bt1490*), and *btuH2* (*bt1956*). All homologs identified across all three searches with E values of ≤0.03 were designated as *btuH* homologs. All downstream comparative genomic analyses focused on *btuH* homologs specifically in *Bacteroidetes*.

### Identification of *btuH* operons.

Previously published operon predictions were used to establish whether *btuB* and *btuH* homologs were present in the same or different operons (12).

### Phylogenetic tree of gut *Bacteroidetes*.

All *Bacteroidetes* strains from the set of 313 genome sequenced gut species were used to create a phylogenetic tree based on all shared proteins. E. coli K-12 ER3466 was used as an outgroup. The tree was created using the PATRIC database (38) full tree method with maximum-likelihood RaxML (39). *btuH* homologs were identified as described above and manually mapped onto the tree.

### Identification of *btuH* and PKD domains.

For each of the *btuH* homologs identified, NCBI BLASTX was used to identify additional N-terminal domains, with an E value cutoff of 1E–3. Specifically, the sequence for B. thetaiotaomicron VPI-5482 *btuG2* (*bt1954*) was used to search for β-propeller domains and the B. thetaiotaomicron VPI-5482 *btuH2* sequence was used to search for PKD domains (bp 91 to 294 for PKD1 and bp 364 to 540 for PKD2).

### Correlations between biosynthesis and transport.

To assess the relationship between vitamin B_12_ biosynthetic capacity and *btuH*, *btuH* homologs were designated as described above. Two independent data sets were used to assess vitamin B_12_ biosynthetic capacity, as defined in the original publications (6, 12), and the results are reported separately. Bacterial strains were manually curated to identify strains that are native to animal gastrointestinal tracts.

### Immunoprecipitation and crystallographic studies.

Methods for immunoprecipitation and crystallography are provided in the supplemental material.

### Data availability.

The mass spectrometry proteomics data have been deposited to the ProteomeXchange Consortium via the PRIDE (40) partner repository with the data set identifier PXD015052. Coordinates and structure factors have been deposited in the Protein Data Bank under accession code 7BIZ.

10.1128/mBio.02845-21.1 TEXT S1 Supplemental materials and methods for protein purification, immunoprecipitation, protein structure determination, and protein structure analysis. Download Text S1, DOCX file, 0.02 MB. Copyright © 2022 Putnam et al. 2022 Putnam et al. https://creativecommons.org/licenses/by/4.0/ This content is distributed under the terms of the Creative Commons Attribution 4.0 International license.
