# Supplementary figures and images for: Gut Commensal Bacteroidetes Encode a Novel Class of Vitamin B12-Binding Proteins
Source: mBio. 2022 Mar 1;13(2):e02845-21. doi: 10.1128/mbio.02845-21 (PMC8941943; doi:10.1128/mbio.02845-21)

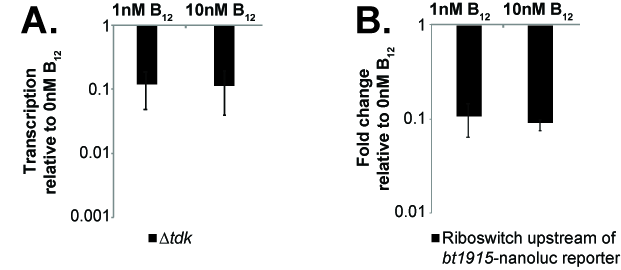

Supplement: FIG S1 [file mbio.02845-21-sf001.tif]

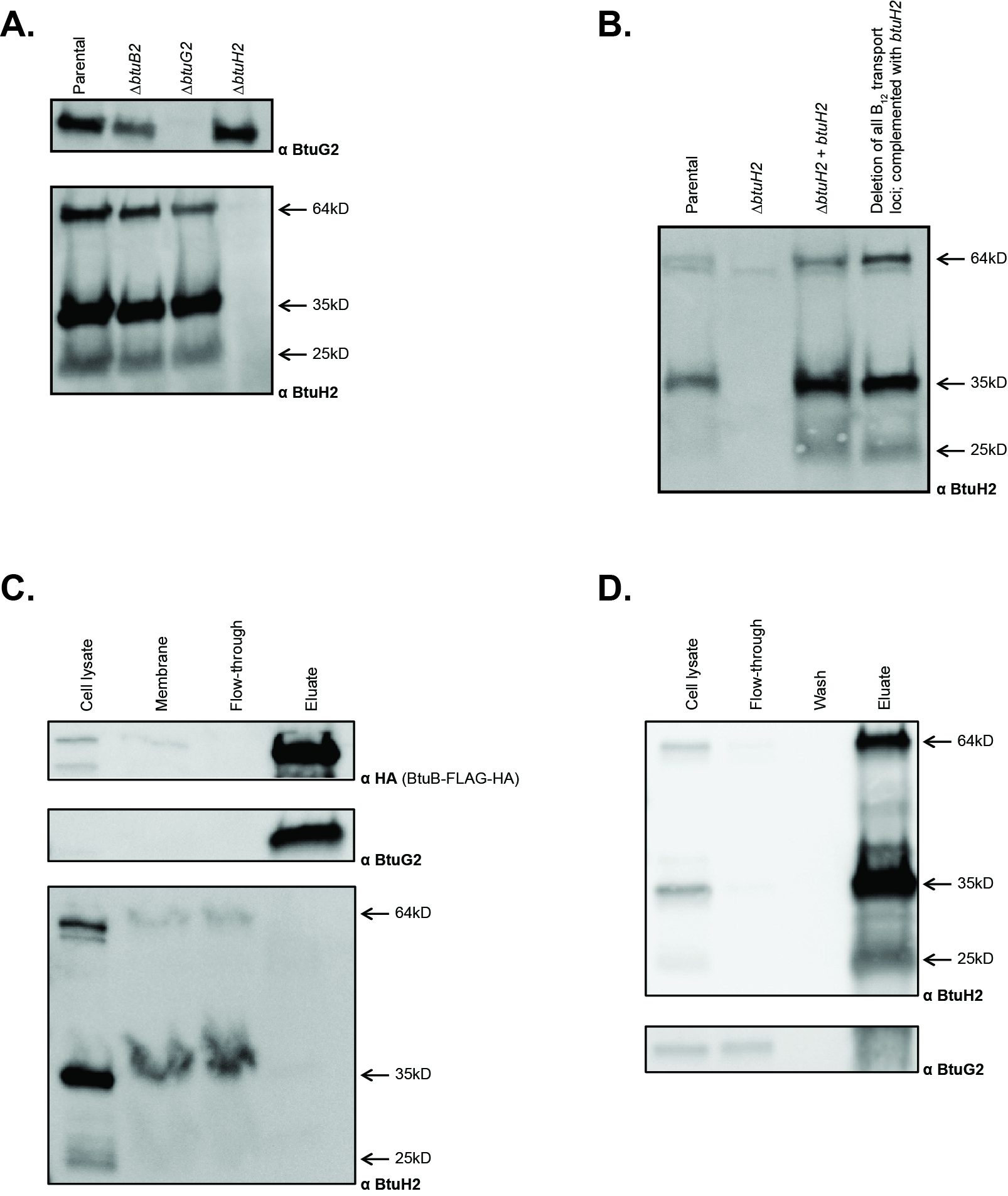

Supplement: FIG S2 [file mbio.02845-21-sf002.tif]

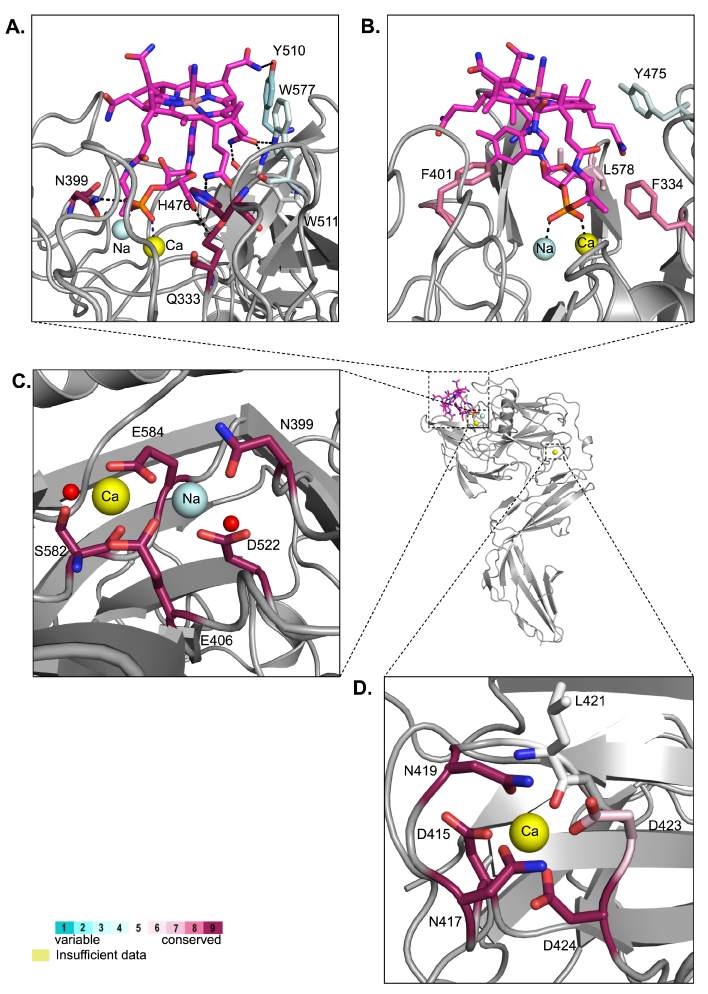

Supplement: FIG S3 [file mbio.02845-21-sf003.tif]

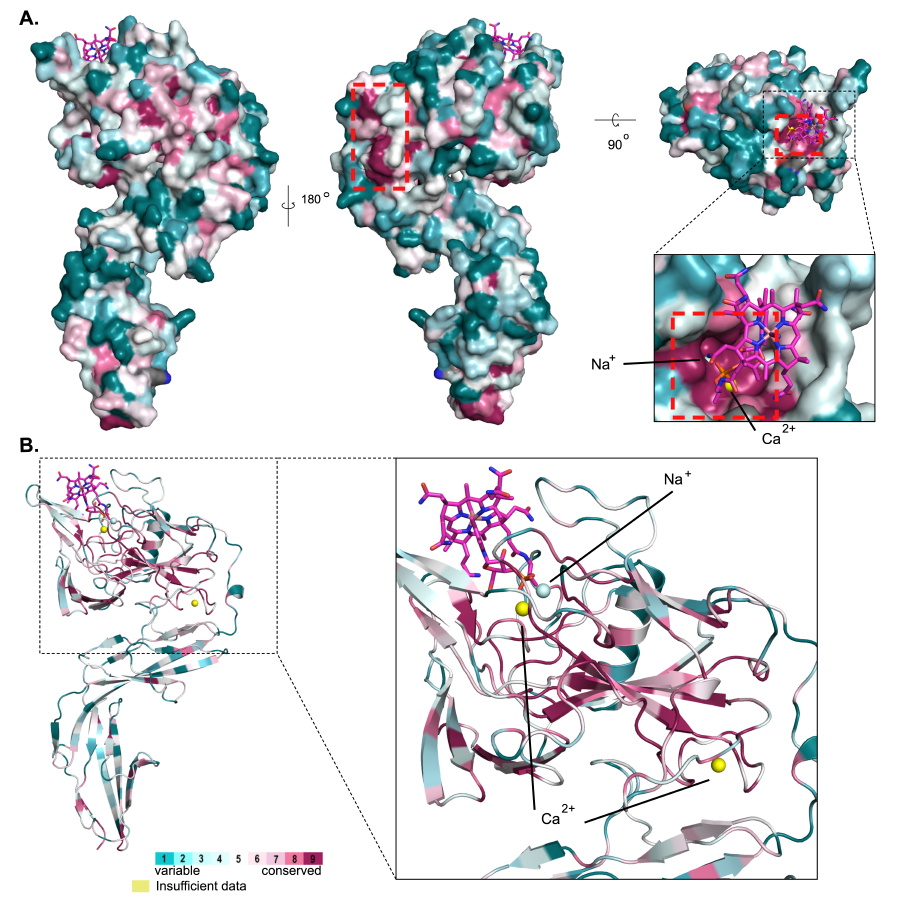

Supplement: FIG S5 [file mbio.02845-21-sf005.tif]

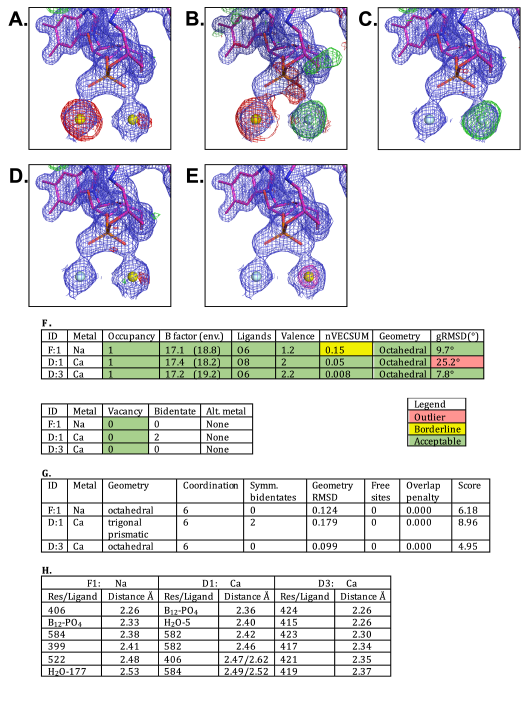

Supplement: FIG S4 [file mbio.02845-21-sf004.tif]

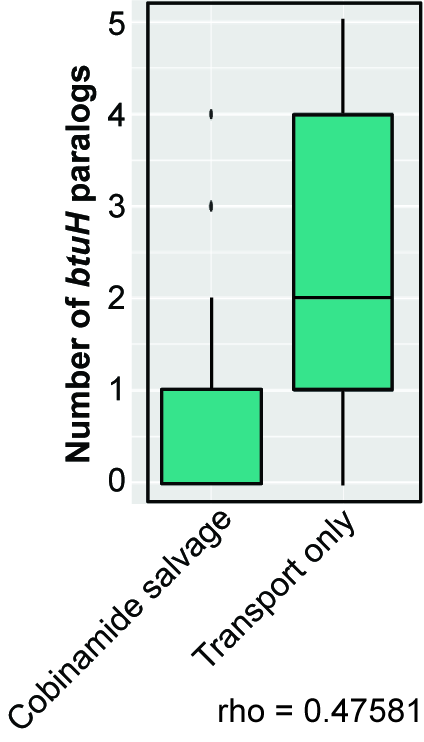

Supplement: FIG S6 [file mbio.02845-21-sf006.tif]
