# Supplementary material for: Gut Commensal Bacteroidetes Encode a Novel Class of Vitamin B12-Binding Proteins
Source: mBio. 2022 Mar 1;13(2):e02845-21. doi: 10.1128/mbio.02845-21 (PMC8941943; doi:10.1128/mbio.02845-21)
Supplement: TABLE S2 [file mbio.02845-21-st002.docx]

**Table S2. Crystallographic tables**

**Table S2A. X-ray crystallographic data collection and refinement statistics.**

|  | **B12-BtuH (103-593*)**  **Co-SAD** | **B12-BtuH (103-593*)**  **high-resolution** |
| --- | --- | --- |
| **Data collection** |  |  |
| Beam | Bruker Metaljet D2 | DLS i24 |
| Wavelength | 1.34 | 0.96864 |
| Space Group | P3121 | P3121 |
| Cell dimensions |  |  |
| a,b,c (Å) | 127.57, 127.57, 135.59 | 128.05, 128.05, 135.05 |
| a,b,g (°) | 90, 90, 120 | 90, 90, 120 |
| Molecules in AU | 2 | 2 |
| Resolution range(Å) | 24.96-1.9 (1.93-1.90) | 85.74-1.54 (1.57-1.54) |
| I/ sI | 15.3 (2.2) | 13.2 (0.9) |
| Completeness (%) | 100 (100) | 100 (100) |
| Redundancy | 66.9 (30.4) | 10.8 (10.7) |
| Rpim (%) | 6.8 (54.4) | 2.6 (70.5) |
| CC (1/2) | 0.994 (0.558) | 0.99 (0.58) |
| Anomalous completeness | 100 (100) | 100 (100) |
| Anomalous redundancy | 33.6 (15) | 5.5 (5.5) |
| **Phasing** |  |  |
| SOLVE FOM | 0.16 | - |
| Sites found [expected] | 2 [2] | - |
| **Refinement** |  |  |
| Resolution (Å) | - | 1.53-85.70 |
| Rwork/Rfree‡ (%) | - | 16.49/19.16 |
| Reflections | - | 373,860 |
| No. Atoms | - |  |
| Protein | - | 7,432 |
| B12 | - | 186 |
| B-factors (Å2) | - |  |
| Protein | - | 31 |
| B12 | - | 24.6 |
| Rmsd | - |  |
| Bond lengths (Å) | - | 0.009 |
| Bond Angles (°) | - | 1.52 |
| Molprobity clashscore | - | 2.95 |
| Ramachandran plot | - |  |
| Favoured (%) | - | 97.12 |
| Disallowed (%) | - | 0.11 |
| PDB code | - | 7BIZ |

**Table S2B. B_12_ binding domain of BtuH2 structure shows very low resemblance with any previously deposited protein.**

Summary of a DALI comparison between the BtuH2 binding domain X-ray crystal structure and those in the Protein Data Bank.

| **PDB-Chain** | **Z-score** | **Rmsd**  **(Å)** | **lali** | **nres** | **%id** | **Molecule description** |
| --- | --- | --- | --- | --- | --- | --- |
| 5xnr-A | 7.4 | 2.7 | 109 | 385 | 8 | ALYQ |
| 4txw-A | 7.2 | 3.5 | 131 | 159 | 11 | HYALURONOGLUCOSAMINIDASE |
| 1tvg-A | 7.2 | 3.2 | 117 | 136 | 11 | LOC51668 PROTEIN |
| 4a3z-A | 7.2 | 3.2 | 121 | 136 | 7 | ALPHA-N-ACETYLGLUCOSAMINIDASE FAMILY PROTEIN |
| 2yc2-B | 7.2 | 3.3 | 115 | 132 | 10 | INTRAFLAGELLAR TRANSPORT PROTEIN 25 |
| 2yc4-B | 7.1 | 3.3 | 115 | 131 | 11 | INTRAFLAGELLAR TRANSPORT PROTEIN 25 |
| 2yc4-A | 7.1 | 3.2 | 113 | 131 | 11 | INTRAFLAGELLAR TRANSPORT PROTEIN 25 |
| 2jda-B | 7.0 | 2.9 | 115 | 142 | 8 | YECBM32 |
| 5zu5-A | 7.0 | 2.9 | 116 | 493 | 7 | ALGINATE LYASE |
| 6k0v-D | 7.0 | 3.2 | 111 | 562 | 12 | ALPHA-1,3-GLUCANASE |
| 4a6o-B | 7.0 | 3.3 | 122 | 144 | 8 | ALPHA-N-ACETYLGLUCOSAMINIDASE FAMILY PROTEIN |
| 2w3j-A | 7.0 | 3.2 | 106 | 137 | 12 | CARBOHYDRATE BINDING MODULE |
| 2yc2-A | 7.0 | 3.0 | 111 | 132 | 11 | INTRAFLAGELLAR TRANSPORT PROTEIN 25 |
| 2jda-A | 6.9 | 2.8 | 115 | 139 | 7 | YECBM32 |
| 2jd9-A | 6.9 | 2.9 | 115 | 139 | 9 | YECBM32 |
| 5zu6-A | 6.9 | 2.8 | 112 | 152 | 7 | CBM32 DOMAIN |
| 2w1w-A | 6.9 | 3.6 | 113 | 130 | 16 | LIPOLYTIC ENZYME, G-D-S-L |
| 2dck-A | 6.9 | 2.8 | 102 | 320 | 9 | XYLANASE J |
| 1ux7-A | 6.9 | 2.8 | 103 | 119 | 13 | ENDO-1,4-BETA-XYLANASE D |
| 6k0p-A | 6.9 | 3.2 | 111 | 559 | 12 | ALPHA-1,3-GLUCANASE |
| 2dcj-B | 6.9 | 3.3 | 106 | 324 | 9 | XYLANASE J |
| 1w0n-A | 6.9 | 3.0 | 105 | 120 | 12 | ENDO-1,4-BETA-XYLANASE D |
| 5opj-A | 6.8 | 2.8 | 103 | 772 | 10 | RHAMNOGALACTURONAN LYASE |
| 6kka-A | 6.8 | 3.4 | 107 | 327 | 9 | ENDO-1,4-BETA-XYLANASE |
| 2w47-A | 6.8 | 3.4 | 110 | 135 | 13 | LIPOLYTIC ENZYME, G-D-S-L |
| 6kjl-B | 6.8 | 3.3 | 106 | 327 | 9 | ENDO-1,4-BETA-XYLANASE |
| 2dcj-A | 6.8 | 3.5 | 108 | 326 | 10 | XYLANASE J |
| 4gwj-A | 6.8 | 3.3 | 118 | 142 | 6 | PLATELET AGGREGATION FACTOR SM-HPAF |
| 3le0-A | 6.8 | 3.3 | 118 | 142 | 6 | PLATELET AGGREGATION FACTOR SM-HPAF |
| 3lek-A | 6.8 | 3.3 | 118 | 141 | 6 | PLATELET AGGREGATION FACTOR SM-HPAF |
| 4a6o-A | 6.7 | 3.2 | 120 | 144 | 8 | ALPHA-N-ACETYLGLUCOSAMINIDASE FAMILY PROTEIN |
| 5msx-C | 6.7 | 3.0 | 115 | 440 | 6 | PUTATIVE ENDO-1,4-BETA-XYLANASE |
| 4zz8-A | 6.7 | 3.1 | 113 | 132 | 9 | GLUCANASE/CHITOSANASE |
| 4zz5-B | 6.7 | 3.2 | 114 | 131 | 10 | GLUCANASE/CHITOSANASE |
| 4zz8-B | 6.7 | 3.2 | 114 | 131 | 10 | GLUCANASE/CHITOSANASE |
| 2vzr-B | 6.7 | 3.1 | 105 | 126 | 12 | EXO-BETA-D-GLUCOSAMINIDASE |
| 2w87-B | 6.7 | 3.6 | 112 | 138 | 6 | ESTERASE D |
| 2w1w-B | 6.7 | 3.1 | 106 | 132 | 12 | LIPOLYTIC ENZYME, G-D-S-L |
| 6k0u-A | 6.7 | 3.2 | 110 | 559 | 12 | ALPHA-1,3-GLUCANASE |
