# Supplementary material for: Gut Commensal Bacteroidetes Encode a Novel Class of Vitamin B12-Binding Proteins
Source: mBio. 2022 Mar 1;13(2):e02845-21. doi: 10.1128/mbio.02845-21 (PMC8941943; doi:10.1128/mbio.02845-21)
Supplement: TEXT S1 [file mbio.02845-21-s0001.docx]

**Supplemental Materials and Methods**

***Protein purification***

BtuH2-His_10_ was expressed from the pET21 vector in *E. coli* BL21 Rosetta (DE3). Expression was induced for log-phase cultures using 0.5mM IPTG at 37˚C for 3 hours. Cells were harvested by centrifugation, lysed using BugBuster (Millipore Sigma), and lysate was incubated with Ni-NTA agarose resin (Qiagen) for one hour. Resin was then washed with 50mM NaH_2_PO_4_, 300mM NaCl, 20mM imidazole, pH 7.4 prior to elution of protein from resin using 50mM NaH_2_PO_4_, 300mM NaCl, 250mM imidazole, pH 7.4.

***Anti-BtuH immunoprecipitation***

*B. thetaiotaomicron* parental strain was grown to mid-log phase in minimal medium. Cells were lysed in 50mM Tris pH 7.4, 100mM NaCl, 1% Triton X-100, 0.1% SDS, 0.5% sodium deoxycholate, with cOmplete EDTA-free protease inhibitor (Sigma #11873580001) for 30 min at room temperature with gentle mixing. Protein A Dynabeads (Thermo Fisher #10001D) were washed with the same lysis buffer prior to incubation with rabbit anti-BtuH2 polyclonal antibody serum for 60 min at room temperature. Beads were then washed and added to cell lysate and incubated 4˚C with gentle mixing overnight. Beads were washed extensively with a high salt variation of the lysis buffer (1M NaCl) prior to elution with 4x Laemmli buffer at 95˚C and immunoblotting for BtuH2 and BtuG2.

***Anti-HA immunoprecipitation***

The *B. thetaiotaomicron* parental strain was modified to place cobamide transport locus 2 under control of the constitutive P1E6 promoter (Whitaker et al., 2017), and to encode a C-terminal FLAG-HA tag for BtuB2. *B. thetaiotaomicron* P1E6-locus 2 *btuB2-FLAG-HA* was then grown in minimal medium to mid-log phase. Cells were pelleted, resuspended in TSB (20mM Tris, 300mM NaCl, pH 8.0), and lysed by sonication. Samples were filter sterilized to remove unlysed cells and the membrane fraction was pelleted by ultracentrifugation at 100,000x g for 1 hr at 4˚C. Membrane pellets were resuspended in 1.5% w/v lauryldimethylamine-*N*-oxide (LDAO) in TSB and incubated with gentle stirring overnight at 4˚C. Samples were then spun at 100,000x g for 1 hr and supernatant was incubated with anti-HA resin (Thermo #26180Y) at 4˚C overnight. Resin was washed extensively in TSB prior to elution using 4x Laemmli buffer at 95˚C and immunoblotting for HA (to detect BtuB2-FLAG-HA), BtuG2, and BtuH2.

***Cloning, expression, and purification of BtuH2 for crystallographic studies***

Crystallization experiments using the mature BtuH2-His_10_ peptide (lacking the signal peptide predicted by SignalP [Almagro et al., 2019] consisting of 17 amino acids) proved to be unsatisfactory. Since BtuH2 encodes three N-terminal PKD domains, new crystallization experiments were attempted by removing the first 102 amino acids corresponding to the signal sequence and the most N-terminal PKD domain. Primers (Eurofins) were designed to amplify the desired coding region (amino acids 103 to 593). Restriction sites *Nco*I and *Xho*I were included in the primers to allow the cloning of the fragment into the plasmid pET28, generating a C-terminal fusion protein with the His_6_ tag present in plasmid sequence. The cloned fragment was Sanger sequenced (Eurofins Genomics) to verify the absence of any mutation. Transformation of the final plasmid into BL21 (DE3) competent cells was achieved using heat shock. Cells were grown in LB plus kanamycin (50 µg/mL) at 37˚C (180 rpm) and the expression was triggered with 0.4mM of isopropyl β-D-1-thiogalactopyranoside after the culture reached an optical density (OD) of 0.6 at 600 nm. Cultures were grown for an additional 16-20 hours at 16˚C, spun down for 30 minutes at 9,000x g in a JLA8.100 rotor, resuspended in 20 mm Tris-HCl buffer, pH 7.8, containing 300 mm NaCl (TSB) and stored at -20˚C until processed. Cells were lysed in a cell disrupter (Constant Systems 0.75 kW operated at 20-23,000 psi; 1 pass) and centrifuged at 43,500x g (JA25.50 rotor) for 30 minutes. The supernatant was loaded onto a 3 mL nickel column previously equilibrated with 5 column volumes (CV) of wash buffer (TSB containing 25mM imidazole) and then washed with 15-20 CV of the same buffer. Finally, proteins were eluted with 3 CV of wash buffer supplemented with a final concentration of 250 mM imidazole. The eluted proteins were then concentrated to ~3-4 mL using an Amicon Ultra-15 Centrifugal Filter Unit (30kDa cut-off; Millipore) and further purified by size-exclusion chromatography (Superdex 200 16/600, GE healthcare) using 10 mM HEPES pH 7.5, 100 mM NaCl. The purity of the protein was assessed by SDS-PAGE.

***Crystallization and structure determination***

Protein was concentrated to 20 mg/mL and B_12_ dissolved in water was added to aliquots to a molar ratio of ~ 1.5 (B_12_/BtuH2). Sitting drop vapor diffusion crystallization trials were set up with a Mosquito Crystallization robot (TTP Labtech) using the commercial screens JCSG+, Structure, Morpheus, PACT and Index (Molecular Dimensions and Hampton Research). Screens were performed at 20˚C. An initial hit with hexagonal rods/blocks with a distinctly red color was observed in Morpheus condition B1 (0.1M Halogens (NaF, NaBr and NaI) 0.1M imidazole, 0.1 MES monohydrate acid pH6.5 and 30% precipitant mix 1 (40% v/v PEG500 MME, 20% w/v PEG 20000)). After optimization, crystals were flash-frozen in liquid nitrogen directly from the crystal drop. Diffraction data were collected at 100 K at an in-house source Bruker liquid METALJET D2 (high redundancy dataset for Co-SAD) and at Diamond Light Source beamline i24 (high resolution dataset). Data from the home source was processed using SAINT (Bruker, 2012). The space group was determined with POINTLESS (Evans, 2006) and merged using AIMLESS (Evans et al., 2013). Phenix AutoSol (Adams et al., 2010; Terwilliger et al., 2009) was used to solve the phase problem using the anomalous scattering produced by the presence of the cobalt atom from B_12_ (cobalt-based single-wavelength anomalous diffraction). The preliminary model was improved using AutoBuild (Terwilliger et al., 2008) followed by rounds of manual building in Coot (Emsley et al., 2005) and refinement with Phenix (Afonine et al., 2012). The high-resolution dataset was processed with DIALS (Winter et al., 2018), the space group determined with POINTLESS (Evans, 2006) and merged using AIMLESS (Evans et al., 2013). Molecular replacement in Phaser (McCoy et al., 2007) was used to solve the phase problem for the high-resolution dataset, with chain A from the above mentioned Co-SAD experiment used as the search model. The model was manually improved using Coot (Emsley et al., 2004) and refined with Phenix (Afonine et al., 2012). During refinement the scale factor for x-ray stereochemistry weights was set to 0.8 and TLS groups were used, determined automatically within Phenix. MolProbity (Chen et al., 2010) was used to validate protein geometry and PyMOL (PyMOL molecular graphics system, version 1.8, Schrödiner, LLC) for the visualization of the protein structure. X-ray crystallographic data collection and refinement statistics are listed in Table S2.

***Structural analysis***

For metal analysis, the CheckMyMetal web server (Zheng et al., 2014) and the tool METALizer from the portal ProteinsPlus (Fahrrolfes et al., 2017) were used. To calculate the evolutionary conservation of the amino acid positions, the phylogenetic relations of 150 homologous sequences were analyzed and color coded attending to their conservation value using the ConSurf server (Landau et al., 2005; Glaser et al., 2003). The multiple sequence alignment was built using MAFFT (Katoh et al., 2013), collecting the homologues from UNIREF90 (Suzek et al., 2015). The homolog search algorithm used was HMMER (Mistry et al., 2013) (HMMER E-value: 0.0001) with 1 iteration and a maximal and minimal percentage of identity for homologs of 95% and 35% respectively. The search model corresponded to chain A from the structure model. To assess protein-ligand interactions, Pymol (PyMOL molecular graphics system, version 1.8, Schrödiner, LLC), the fully automated protein–ligand interaction profiler PLIP (Salentin et al., 2015) and LigPlot^+^ software packages were used (Laskowski et al., 2011). The DALI server (Holm, 2020) was used for comparing the BtuH2 structure against those in the Protein Data Bank (PDB). The coordinates for the DALI analysis comprised only the B_12_ binding domain (residues 307-592).

**Supplemental References:**

Adams PD, Afonine PV, Bunkoczi G, Chen VB, Davis IW, Echols N, et al. PHENIX: a comprehensive Python-based system for macromolecular structure solution. Acta Crystallogr D Biol Crystallogr. 2010;66(Pt 2):213-21.

Afonine PV, Grosse-Kunstleve RW, Echols N, Headd JJ, Moriarty NW, Mustyakimov M, et al. Towards automated crystallographic structure refinement with phenix.refine. Acta Crystallogr D Biol Crystallogr. 2012;68(Pt 4):352-67.

Almagro Armenteros JJ, Tsirigos KD, Sonderby CK, Petersen TN, Winther O, Brunak S, et al. SignalP 5.0 improves signal peptide predictions using deep neural networks. Nat Biotechnol. 2019;37(4):420-3.

Bruker. SAINT. Madison, Wisconsin, USA: Bruker AXS Inc.; 2012.

Chen VB, Arendall WB, 3rd, Headd JJ, Keedy DA, Immormino RM, Kapral GJ, et al. MolProbity: all-atom structure validation for macromolecular crystallography. Acta Crystallogr D Biol Crystallogr. 2010;66(Pt 1):12-21.

Emsley P, Cowtan K. Coot: model-building tools for molecular graphics. Acta Crystallogr D Biol Crystallogr. 2004;60(Pt 12 Pt 1):2126-32.

Evans P. Scaling and assessment of data quality. Acta Crystallogr D Biol Crystallogr. 2006;62(Pt 1):72-82.

Evans PR, Murshudov GN. How good are my data and what is the resolution? Acta Crystallogr D Biol Crystallogr. 2013;69(Pt 7):1204-14.

Fahrrolfes R, Bietz S, Flachsenberg F, Meyder A, Nittinger E, Otto T, et al. ProteinsPlus: a web portal for structure analysis of macromolecules. Nucleic Acids Res. 2017;45(W1):W337-W43.

Glaser F, Pupko T, Paz I, Bell RE, Bechor-Shental D, Martz E, et al. ConSurf: identification of functional regions in proteins by surface-mapping of phylogenetic information. Bioinformatics. 2003;19(1):163-4.

Holm L. DALI and the persistence of protein shape. Protein Sci. 2020;29(1):128-40.

Katoh K, Standley DM. MAFFT multiple sequence alignment software version 7: improvements in performance and usability. Mol Biol Evol. 2013;30(4):772-80.

Landau M, Mayrose I, Rosenberg Y, Glaser F, Martz E, Pupko T, et al. ConSurf 2005: the projection of evolutionary conservation scores of residues on protein structures. Nucleic Acids Res. 2005;33(Web Server issue):W299-302.

Laskowski RA, Swindells MB. LigPlot+: multiple ligand-protein interaction diagrams for drug discovery. J Chem Inf Model. 2011;51(10):2778-86.

McCoy AJ, Grosse-Kunstleve RW, Adams PD, Winn MD, Storoni LC, Read RJ. Phaser crystallographic software. J Appl Crystallogr. 2007;40(Pt 4):658-74.

Mistry J, Finn RD, Eddy SR, Bateman A, Punta M. Challenges in homology search: HMMER3 and convergent evolution of coiled-coil regions. Nucleic Acids Res. 2013;41(12):e121.

Salentin S, Schreiber S, Haupt VJ, Adasme MF, Schroeder M. PLIP: fully automated protein-ligand interaction profiler. Nucleic Acids Res. 2015;43(W1):W443-7.

Suzek BE, Wang Y, Huang H, McGarvey PB, Wu CH, UniProt C. UniRef clusters: a comprehensive and scalable alternative for improving sequence similarity searches. Bioinformatics. 2015;31(6):926-32.

Terwilliger TC, Adams PD, Read RJ, McCoy AJ, Moriarty NW, Grosse-Kunstleve RW, et al. Decision-making in structure solution using Bayesian estimates of map quality: the PHENIX AutoSol wizard. Acta Crystallogr D Biol Crystallogr. 2009;65(Pt 6):582-601.

Terwilliger TC, Grosse-Kunstleve RW, Afonine PV, Moriarty NW, Zwart PH, Hung LW, et al. Iterative model building, structure refinement and density modification with the PHENIX AutoBuild wizard. Acta Crystallogr D Biol Crystallogr. 2008;64(Pt 1):61-9.

Whitaker WR, Shepherd ES, Sonnenburg JL. Tunable Expression Tools Enable Single-Cell Strain Distinction in the Gut Microbiome. Cell. 2017;169(3):538-46 e12.

Winter G, Waterman DG, Parkhurst JM, Brewster AS, Gildea RJ, Gerstel M, et al. DIALS: implementation and evaluation of a new integration package. Acta Crystallogr D Struct Biol. 2018;74(Pt 2):85-97.

Zheng H, Chordia MD, Cooper DR, Chruszcz M, Muller P, Sheldrick GM, et al. Validation of metal-binding sites in macromolecular structures with the CheckMyMetal web server. Nat Protoc. 2014;9(1):156-70.
